# Revisiting Active Site Quantification in CO_2_ Electroreduction: The Case for CO Displacement

**DOI:** 10.1021/acsenergylett.5c01642

**Published:** 2025-08-12

**Authors:** Yuxiang Zhou, Benjamin Bowers, Alexander Bagger, Guangmeimei Yang, Ludmilla Steier, Mary P. Ryan, Ifan E. L. Stephens

**Affiliations:** † Department of Materials, Imperial College London, South Kensington Campus, Exhibition Road, London, SW7 2AZ, United Kingdom; ‡ Department of Physics, Technical University of Denmark, Kongens Lyngby 2800, Denmark; § Department of Chemistry, Imperial College London, Molecular Sciences Research Hub, 82 Wood Lane, W12 0BZ, London, United Kingdom; ∥ Department of Chemistry, Oxford University, 12 Mansfield Road, Oxford, OX1 3TA, United Kingdom

## Abstract

The selectivity and geometric current density of copper-based electrodes for electrochemical CO_2_ reduction (CO_2_RR) have been significantly improved, yet research is striving to improve the intrinsic activity of these materials. The accurate quantification of active sites is vital to benchmark the intrinsic activity of the catalysts for electrochemical CO_2_ reduction to facilitate activity improvements. Herein, we propose a method to determine the active sites using CO displacement in potassium phosphate buffer at 10 °C. Comparing this method with the electrochemical surface area (ECSA), measured by double-layer capacitance, the most used technique in this field, we demonstrate that CO displacement provides a much more accurate quantification of the number of active sites. By normalizing current density vs the CO displacement active sites, we find electropolished copper foil has the highest intrinsic activity towards CO_2_RR. We also reveal there is a clear relationship between surface roughness and chained products.

## Graphical abstract

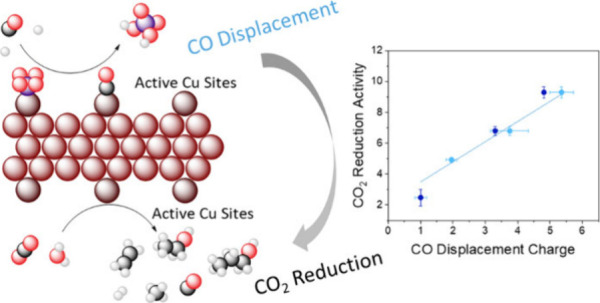

Electrochemical CO_2_ reduction (CO_2_RR) provides a strategy to achieve low carbon fuels and chemical feedstock. To date, copper (Cu) is the only monometallic catalyst that can yield, high value, C_2+_ molecules at high faradaic efficiencies (FE) due to its suitable binding energy towards key intermediates *CO and *H.^1^ Significant strides have been made in altering the selectivity of these Cu-based catalysts by tuning the nanostructure,^2–5^ composition,^6–16^ utilizing facet effects^17,18^ and electrolyte effects.^19–24^ As a result, the FE towards valuable products ethylene and ethanol have been reported to reach up to 87%^25^ and 52%^26^ respectively.

Such highly selective Cu-based CO_2_RR catalysts still suffer from low intrinsic activity and poor stability. To the best of our knowledge, electropolished polycrystalline Cu still shows the highest CO_2_RR intrinsic activity, despite forming 16 different CO_2_RR products.^27–29^ Moreover, Cu tends to reconstruct and degrade under reaction conditions.^30–33^ Therefore, to scale up this reaction, a catalyst with similar intrinsic CO_2_RR activity to polycrystalline Cu, but much better selectivity and long-term stability must be found.^34–36^ The discovery of such a catalyst would be strongly accelerated if we had improved our understanding of the factors controlling CO_2_ reduction and in particular improved methods to measure the intrinsic catalytic activity.

An accurate estimation of intrinsic activity is crucial to benchmark a catalyst performance and understand the CO_2_RR kinetics and, in turn, the mechanism. In electrocatalysis, intrinsic activity of a catalyst is and can only be evaluated by the turnover frequency (TOF), the number of moles of reactant converted per active site per unit time.^29^ In practice, partial current density normalised by the electrochemical active surface area (ECSA) is often used to represent the TOF. Double layer capacitance (C_dl_) is the most widely used method for measuring ECSA of Cu-based materials.^37^ C_dl_ consists of cyclic voltammetry (CV) under different scan rates in the potential window without the Faradaic process. The buildup of charge and discharge is measured and related to the capacitance.^38^ However, discrepancies often arise between the ECSA measured by double layer capacitance and other methods, like Pb-under potential deposition (UPD).^37,39^ Additionally, the adsorbed species during C_dl_ measurement remains unclear.^40^ Even on Pt single crystals, a much more well understood surface, the double layer capacitance can easily change by a factor of 2 between different facets, and there are huge discrepancies on the surface area measured by different methods, including CO stripping, H-UPD, Pb-UPD, and double layer capacitance.^41^ Single crystal studies on Cu showed similar issues (Figure S1). Furthermore, there are questions whether non-faradaic capacitance can be related to CO_2_RR active sites, at reaction potential,^42^ as the sites that are active for double layer capacitance measurement might not be also active for CO_2_RR. Therefore, a new method for measuring the CO_2_RR active sites on Cu-based materials is required which allows performance to be more accurately related to intrinsic activity.

In the 1990s, Hori et al. first showed that a charge transfer occurs on a Cu electrode under cathodic conditions in phosphate buffer with CO present. A reproducible and sharp peak at around −0.7 V vs NHE appeared in the CV scan (Figure S2a).^43^ The in situ Fourier-transform infrared spectroscopy (FTIR) studies indicated that this charge transfer process involved the exchange between CO and adsorbed phosphates (HPO_4_^2–^ and PO_4_^3–^) on the Cu surface, which can be termed as the CO displacement reaction.^44,45^ Such process was strongly dependent on the electrolyte pH (Figure S2b). Phosphoric acid is a tritropic acid with p*K*_a_ values of 2.15, 7.21, and 12.35.^46^ In pH 2.15 to 7.21 aqueous solution, the most common CO_2_RR pH range, CO displacement reactions can be described by the following two equations.

1 HPO4*+H++2e−+CO↔H2PO4−+CO*

2 PO4*+2H++3e−+CO↔H2PO4−+CO*

where * denotes adsorbed species. Hori and co-workers’ electrochemistry data showed that the CO displacement peak shifts around 37 mV pH^–1^ in the neutral pH region (Figure S2b),^44^ indicating that eq 2 is the main reaction occurring in pH 2.15 to 7.21 phosphate buffer. A similar trend and pH-dependent voltage slope was also observed by Sebastián-Pascual et al.^46^

Hori’s work additionally showed that the charge transferred during the CO displacement process is independent of CV scan rate, providing the possibility of using this method to estimate the amount of CO_2_RR active sites of Cu-based materials.^45^ Additionally, numerous spectroscopic studies,^47–51^ and DFT calculations coupled with principal component analysis based on the Hori’s^52,53^ CO_2_ reduction results of various Cu single crystals conducted by Bagger et al.^1,54,55^ provide strong evidence that *CO binding energy serves as an effective descriptor for CO_2_ reduction towards more reduced products beyond CO - typically the desired products on Cu surfaces. Therefore, compared with the conventional double layer capacitance method, CO displacement should allow a better quantification of the CO_2_RR active sites on Cu-based materials for >2e^–^ products, under the assumption of no surface reconstruction.

Hori et al. further found a potential-temperature dependence for CO displacement (Figure S2c). Decreasing temperature can enhance the solubility of CO in electrolyte and suppress the hydrogen evolution reaction (HER) and CO reduction reaction (CORR),^45^ allowing a visible peak of the displacement reaction in a CV. Single crystal studies showed both the CO displacement charge and double layer capacitance has a crystalline facet dependence (Figure S3). For double layer capacitance, even on the same facets and under the same electrolyte, significantly different values (C_dl_, μF cm^–2^) were obtained by different groups, as demonstrated by Figure 1 a. Previous studies from Engstfeld et al. also showed that different polishing and cleaning methods on polycrystalline Cu could also lead to different CVs, resulting in varying double layer capacitances.^56^ For CO displacement, Hori et al. showed that on Cu (111), CO does not displace phosphate, i.e., the displacement charge is zero. On the other hand, there is a linear correlation between the displacement charge and the number of all other atoms, including Cu(100) and step sites, as shown in Figure 1b. This correlation is consistent with multiple groups’ observations on Cu single crystals that closely packed Cu(111) is inactive towards CO_2_RR (presumably because CO does not adsorb strongly onto it) while the remaining sites contribute towards CO_2_RR activity.^52,55,57^ However, it has thus far been unclear how these observations on planar extended surfaces translate directly to technologically relevant nanostructured materials.

**Figure 1:**
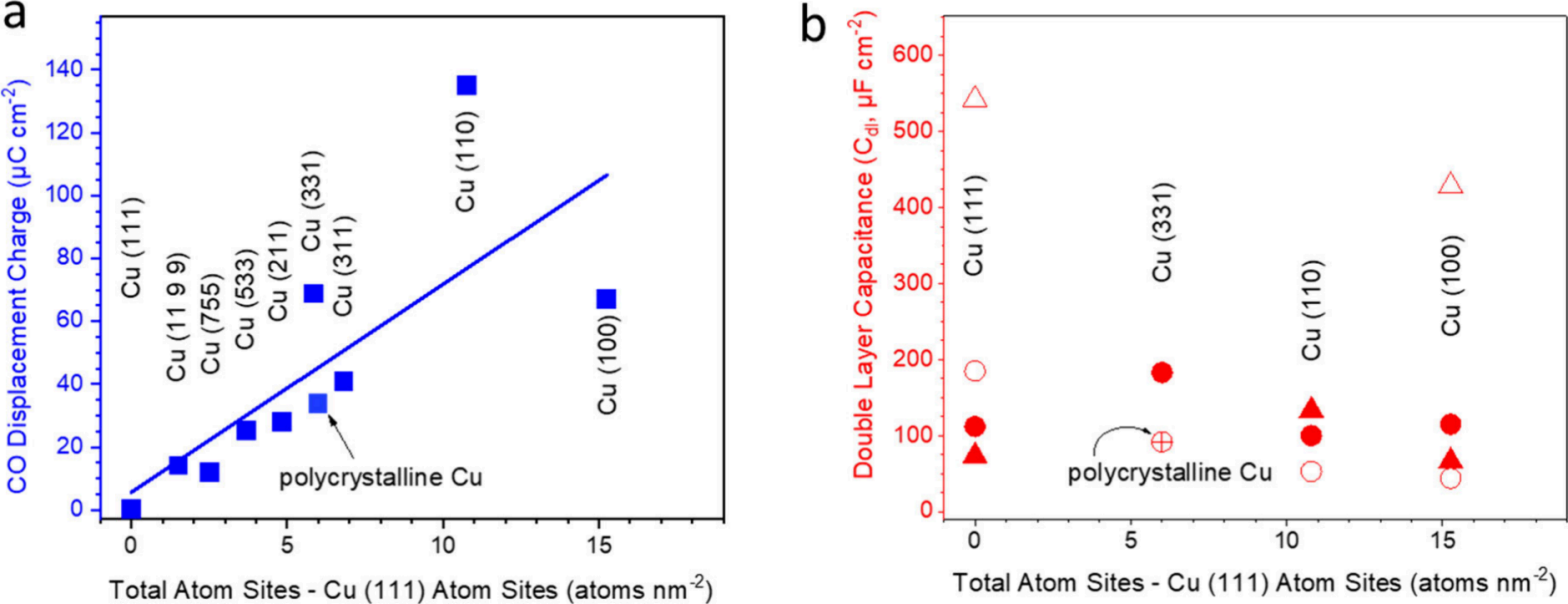
Comparison of the facet dependence of the (a) CO displacement charges in pH 6.8, 0.2 M phosphate buffer, and (b) double layer capacitances. The X axis converts the Cu facets to the densities of Cu atoms ((111) not included). The CVs used for calculating the double layer capacitances and CO displacement charges can be found in Figures S1 and S3, respectively. Data was obtained from Koga, Hori, Hochfilzer, Tiwari, Engstfeld, Raaijman, Le Duff, Schouten et al.^43,44,56,58–64^ .

Herein, we explore the usage of the CO displacement charge as a probe of the number of CO_2_RR active sites on Cu-based nanostructured catalysts, under the assumption of no surface reconstruction; on this basis, we are able to provide a more accurate measure of the intrinsic catalytic activity of Cu-based materials.

We aimed to replicate Hori’s CO displacement experiments in 0.2 M phosphate buffer electrolyte under different temperatures and pH (Figure S4). The experiments were undertaken in an H-cell configuration on an electropolished copper foil electrode; our XRD experiments showed that this foil had a preferential orientation in the (200) plane. The copper foil electrode was electropolished at 2.1 V in 85% H_3_PO_4_ for 5 min. The anolyte was cooled in a jacket beaker and circulated into the cell at 5 mL min^–1^ using a peristaltic pump (Figures S5 and S6). A sustainion anion exchange membrane (AEM) was used to separate the anolyte and catholyte, limiting electrolyte cross over, but allowing heat transfer to cool down the catholyte. The temperature was monitored by an alcohol thermometer. The reaction was completed in 0.2 M K_2_HPO_4_ (pH 9) and 0.1 M K_2_HPO_4_ + 0.1 M KH_2_PO_4_ (pH 6.8), at temperatures of 10, 16 and 20 °C. The copper foil electrode was prereduced in Ar (at −226 μA cm^–2^) until the cell potential increased to around −0.9 V and CVs were swept between −0.5 and −1.1 V vs NHE until a consistent current response formed.^44^ CO was introduced with the electrode held at −0.5 V vs NHE to form the metallic state of copper and the cell was purged for 30 min before repeating the same CV scans as in Ar (see the Supporting Information for a more detailed methodology).

Our results showed a similar trend to Hori et al., with CO displacement peak size decreasing with increasing temperature (Hori et al.^43^ in Figure S2c and this work in Figure S4) and a pH dependence of 32 mV pH^–1^ was observed compared to the value of 37 mV pH^–1^ described by Hori et al.^43^ (Figure S4c). We attribute the lower value of the CO displacement charge across the pH and temperature range in our experiments to differences in the copper foil, electropolishing method, and cells used.^43,45,65^

To assess double layer capacitance and CO displacement for quantifying CO_2_RR active sites, an electropolished copper foil was pretreated by three different ways to change the surface roughness; electropolishing (Figures 2a and S7a), anodization (Figures 2b,c and S7b,c), and electrodeposition (Figure 2d and S7d). The different methods for nanostructuring occurred on different pieces of the same (200) preferred orientation copper foil (Figure 2). All copper foil electrodes were mechanically polished and electropolished before pretreatment. Anodized samples (Figure S7b,c) were prepared by applying 8 mA cm^–2^ to the working electrode Cu foil in 3 M KOH to form Cu­(OH)_2_ nanowires.^66,67^ This was followed by electrochemical reduction at −226 μA cm^–2^ in 0.1 M KHCO_3_. The potential for the anodization increased as nanowires formed. We curtailed the potential to a maximum of 2 V vs Ag/AgCl to avoid delamination of the Cu­(OH)_2_ nanowires. Just before this point, the surface was “fully anodized”. After 1.5 min of anodization and then reduction (Figure 2b), the surface was roughened but had not yet formed the dendritic structure in Figure 2c named “fully anodized”. Copper “flakes” decorated the surface, linked to the dissolution of copper and redeposition,^68^ which was the proposed process for the dendrite formation.^66^ The electrodeposited samples were prepared by applying −0.2 mA cm^–2^ to the working electrode Cu foil in a CuSO_4_, lactic acid and KOH solution, until a total of 3 C charge passed^69^ (Figure S7d). A cubic structure was formed. As before, this was followed by an electrochemical reduction After 3 C of charge passed, the cubes completely covered the copper foil surface.

**Figure 2:**
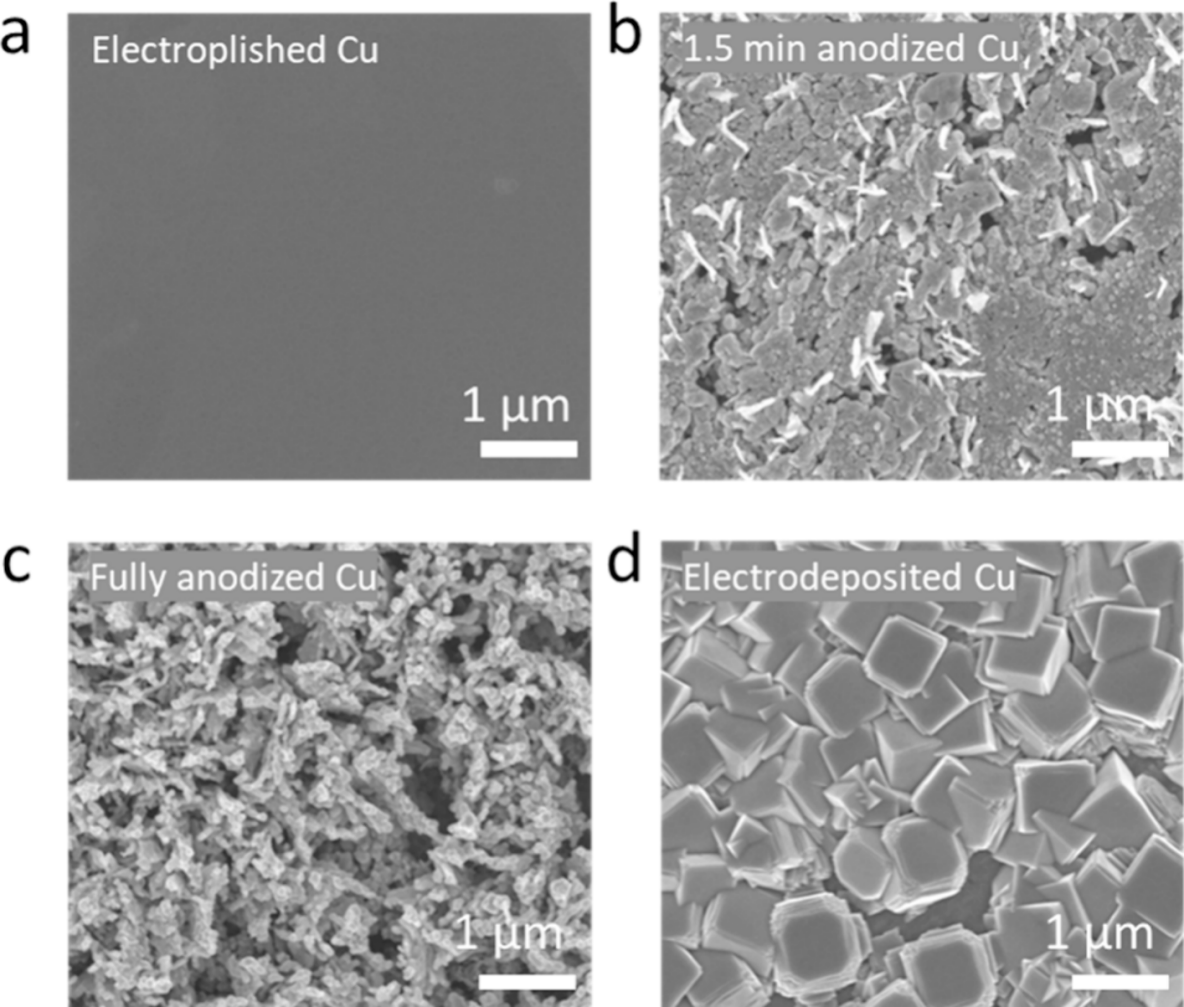
SEM images of the 4 pretreated electrodes. (a) Polycrystalline Cu electropolished at 2.1 V in 85% H_3_PO_4_ for 5 min; (b) 1.5 min anodized Cu at 8 mA cm^–2^ in 3 M KOH, followed by the in situ reduction at −226 μA cm^–2^ in 0.1 M KHCO_3_; (c) Fully anodized Cu at 8 mA cm^–2^ in 3 M KOH followed by the same in situ reduction process; (d) Electrodeposited Cu in a CuSO_4_, lactic acid and KOH solution for 3 C of charge passed followed by the same in situ reduction process. SEM images were taken on a Zeiss Sigma, with 5 kV as the acceleration energy of the incident electron beam.

CO displacement CVs were taken with an H-cell in pH 6.8 buffer (0.1 M K_2_HPO_4_ + 0.1 M KH_2_PO_4_), matching the pH for CO_2_RR (0.1 M KHCO_3_), at 10 °C. As previously discussed, the CO displacement experiment conducted at lower temperature (10 °C) to minimize the influence of HER current, improving the signal to noise ratio, but still does not veer too far from the temperature at which we conduct our catalytic tests. This condition was used for subsequent data analysis and comparison to the CO_2_RR activity. We note that the CO_2_RR is typically performed at room temperature (approximately 20 °C), and in electrolyte with different anion type (HCO_3_^‑^) and cation (K^+^) concentrations. The CO adsorption sites on Cu surfaces are assumed not to be significantly affected by this ∼10 °C temperature and electrolyte-type difference. The redox peaks were caused by the displacement between surface adsorbed CO and phosphate ions. The peak was integrated and highlighted by a blue fill (Figures 3 and S8) (see Supporting Information for further information). This peak was at its most sharp for the electropolished sample (Figure 3a,c), which has a strong preferential orientation along the (200) plane (Figure S9). The more diffuse peaks were observed on the rougher samples, which have a greater contribution in the GIXRD from planes parallel to (111) and (110) (Figure S9), consistent with the corresponding single crystal surfaces probed by Hori et al.^43,45^ The potential for displacement was similar on the electropolished and anodized samples yet occurs at a slightly more negative potential for the electrodeposited sample (Figures 3 and S8). The peak was reproducible for many cycles and absent under Ar (Figures 3a, S10, and S11). Potential hold measurements, where a chronoamperometry measurement was held at a potential just more negative than the displacement peak with and without CO, further confirmed that this current resulted from the CO displacement reaction (Figures 3c and S8). All potential hold experiments were performed at −0.85 V vs NHE, which was more negative than the displacement peak positions from Hori’s single crystal studies (Figure S3)^43,59^ and our own CV experiments (Figure S8). The initial current spike after the introduction of the CO was caused by the displacement with phosphate ions. Thereafter, the current decreases as the CO poisons the surface and diminishes the current due to H_2_ evolution. However, a different magnitude of the CO poisoning effect to the HER current was observed in the repeating experiment shown by Figure 3a. As the HER behaviour of Cu with the presence of CO is complicated process^70^ and beyond the scope of this paper, brief explanations on such feature can be found in SI. Potential hold also allows roughness factor comparisons between the current difference after CO introduction (Figures 3c and S8) and the integrated CV peak (Figures 3 and S8). The potential for hold was lower than the onset for the CORR, as shown by the electrochemistry mass spectrometry measurement (Figure S12).

**Figure 3:**
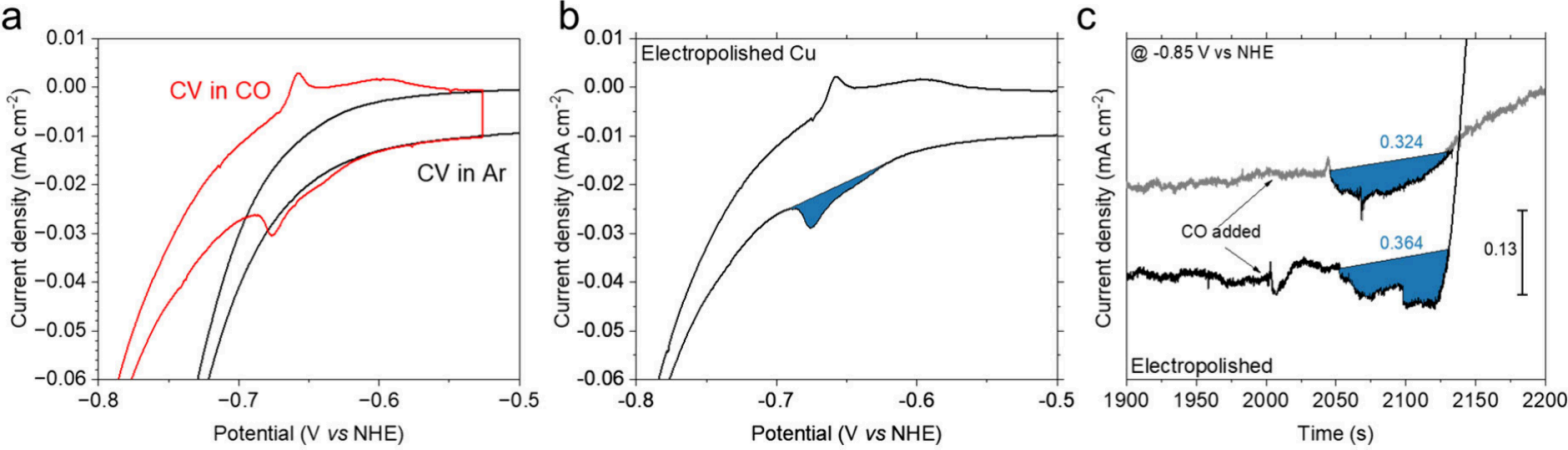
(a) Comparison of CVs for the electropolished copper foil in CO and Ar; (b) CO displacement CV of the electropolished Cu foil with the peak highlighted in the blue fill. The baseline of the peak was manually fitted by using the Origin software. Please see Supporting Information for further details of the peak area integration. CVs in (a) and (b) were conducted with the scan rate of 50 mV s^–1^ at 10 °C in pH 6.8 KH_2_PO_4_ phosphate buffer (0.1 M K_2_HPO_4_ + 0.1 M KH_2_PO_4_) with CO or Ar purged. CVs with different circle number were chosen and presented in both plots; (c) Chronoamperometry curves of the potential hold measurements at −0.85 V vs NHE in pH 6.8 phosphate buffer (0.1 M K_2_HPO_4_ + 0.1 M KH_2_PO_4_) on the electropolished copper foil and repeated on different electrodes.

The electropolished polycrystalline Cu was chosen as the reference and hence have a double layer capacitance roughness factor (RF) and normalized CO displacement charge of 1. The cathodic CO displacement peak area (Figure S8) of the nanostructured Cu was compared with that of the electropolished Cu to calculate the normalised CO displacement charge. The 3rd cycle of each CV scan was integrated, with the baseline of the slope corrected (Figure S13). The increase in current with the introduction of CO in the transient current curve (Figures 3c and S8) was also integrated and compared to the value for electropolished Cu to gauge further comparisons. The double layer capacitance was taken by cycling at various scan rates around 0 V *vs* the RHE in 0.1 M HClO_4_ (Figure S14). The change in discharge current with increasing scan rates for the different electrodes were compared to electropolished Cu to form the roughness factor.

As mentioned, the normalized CO displacement charge and RF for the nanostructured electrodes were calculated against the electropolished electrode (CV integration, light blue, and potential hold, dark blue in Figures 4 and S15). The resulting raw values can be found in Table S1 and plotted in Figures 4 and S15. The nanostructured electrodes were tested toward the CO_2_RR with an H-cell configuration in 0.1 M KHCO_3_ electrolyte at −1.0 V vs RHE for an hour per potential.

**Figure 4:**
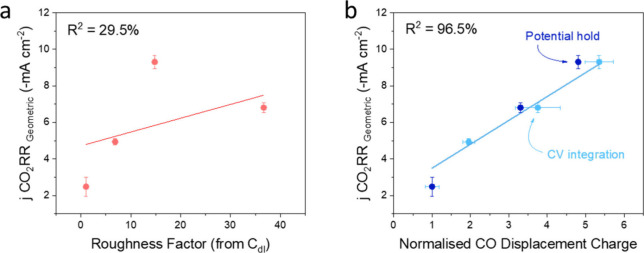
Relationships between the CO_2_ reduction partial current densities normalized to geometric surface area (measured in 0.1 M KHCO_3_, at −1.0 V vs RHE, and in H-cell) and (a) roughness factor measured by double layer capacitance (red), (b) normalised CO displacement charge measured by CV (light blue), and potential hold (dark blue). Error bars were calculated by repeating both the CO displacement reaction and CO_2_RR for different electrodes and comparing the active surface area normalized current density towards CO_2_RR.

As shown by Figure 4a, for the double layer capacitance RF vs the geometric CO_2_RR current density, there is an absence of a linear relationship. Conversely, the normalized CO displacement charge (Figure 4b) follows a linear relationship, well fitted vs the geometric CO_2_RR current density. Another way of illustrating this is through plotting the active surface area normalized current density toward CO_2_RR for CO displacement (CV integration, light blue, and potential hold, dark blue) and double layer capacitance (red) vs the nanostructured electrodes. In this case, a similar value indicates a similar estimated intrinsic activity towards CO_2_RR of all different pretreated Cu-electrodes (Figure S15c). The double layer capacitance significantly overestimates the number of CO_2_RR active sites in this work. The CO displacement, however, neatly normalizes the CO_2_RR current density to the active surface area and deduces that increasing surface roughness through different methods does not improve the intrinsic activity. We propose that both CV integration (Figures 3a and S8a–d) and potential hold measurements (Figures 3b and S8e–h) are required for a more accurate CO_2_RR active sites measurement due to the errors in integrating the CV with a broad peak and likewise, the errors in integrating the transient current increase for electrodes with lower CO_2_RR active sites (Figure 3b). However, as no current active sites or ECSA quantification methods are absolute, in case of these two methods differ, we recommend cross comparing them with the results obtained from other characterization methods, like Pb-UPD, BET, CO-temperature-programmed deposition, and even the morphology analysis from electron microscopies, and consider the other properties of the Cu-based materials synthesized, to make appropriate selections.

The geometric and CO displacement active surface area normalized current densities are compared to other literature values normalized to double layer capacitance, with data adapted from Nitopi et al. (Figure S16).^71^ The current density normalized by CO displacement active surface area for the pre-treated electrodes converge to a greater extent at a higher partial current density than any other literature values. The above results also show that increasing the surface roughness does not improve the activity. Targeting surface roughness primarily affects the reaction selectivity by increasing the number of active sites without enhancing the intrinsic activity of each site. As surface roughness increases, so does the partial current density for oxygenated products, suppressing C_1_ products on the electrode vs C_2_ (Figure S17b). Surface roughness has two possible effects (i) increasing the number of strong binding undercoordinated sites^72,73^ and (ii) effecting changes in mass transport of reactants and pH gradients.^74–76^ It is therefore vital to accurately account for the active sites, not only to gauge the intrinsic activity of novel electrodes but also to accurately elucidate and model the effect of other surface changes on selectivity.

Our findings strongly suggest that closely packed (111) surfaces are inactive on nanostructured Cu surfaces, analogous to earlier studies on Cu single crystals:^57,77–80^ we hence confirm the notion that findings on model extended surfaces can be translated to industrially relevant rough/high surface area Cu.

To conclude, this paper highlights the importance of accurate CO_2_RR active sites measurements for the progression of material design, as research aims to improve the intrinsic activity of Cu and create novel materials towards CO_2_RR. By comparing the CO_2_RR current densities of 4 different nano-structured Cu electrodes normalized by double layer capacitance and CO displacement charge, we have shown CO displacement has distinct advantages over the widely used double layer capacitance method. To incorporate the method widely into the field, we currently recommend always benchmarking against electropolished Cu in pH 6.8 buffer at 10^o^C to gain a clear integration peak and reduce the error of the measurement. Future studies should focus on how variations in the CO displacement temperature, electrolyte pH, Cu working electrode potentials during the CO_2_RR, and exposed Cu facets affect the accuracy of active sites quantification. Furthermore, it is imperative that the (or a) method is developed to allow the measurement of CO_2_RR activity sites in industrially relevant single cells, such as membrane electrode assemblies, before, during, and after CO_2_RR operation.

## Supplementary Material



## Notes

Experimental and data analysis details, data in table format, and additional plots (PDF)
