# Supplementary material for: Revisiting Active Site Quantification in CO2 Electroreduction: The Case for CO Displacement
Source: ACS Energy Lett. 2025 Aug 12;10(9):4324–31. doi: 10.1021/acsenergylett.5c01642 (PMC12442089; doi:10.1021/acsenergylett.5c01642)
Supplement: Supplementary file 1 [file nz5c01642_si_001.pdf]

Revisiting Active Site Quantification in CO_2 Electroreduction: The Case for CO Displacement – Supporting Information.

Yuxiang Zhou [a$], Benjamin Bowers [a$], Alexander Bagger [b], Guangmeimei Yang [c], Ludmilla Steier [d], Mary P. Ryan* [a], Ifan E. L. Stephens* [a].*

[$] Y.Z. and B.B. contributed equally to this work. The sequence of these two authors does not represent the contributions.

[a] Department of Materials, Imperial College London, SW7 2AZ, London, UK

[b] Department of Physics, Technical University of Denmark, Kongens Lyngby 2800, Denmark

[c] Department of Chemistry, Imperial College London, SW7 2AZ, London, UK

[d] Department of Chemistry, Oxford University, 12 Mansfield Road, Oxford, OX1 3TA, UK

Corresponding Author: Ifan E. L. Stephens (i.stephens@imperial.ac.uk); Mary P. Ryan (m.p.ryan@imperial.ac.uk); Ludmilla Steier (ludmilla.steier@chem.ox.ac.uk)

Experimental and data analysis details

CO displacement protocol

‘CV integration’

1. Pre-treat electrode (see main text for details). 0.5 mm thick Cu plate (Alfa Aesar Supratronic®, purity 99.9999%) were used as the working electrode in this work.
2. Begin to cool down the electrolyte in the jacket beaker (takes about an hour).
3. Fabricate H-cell with pre-treated electrode.
4. Purge the airtight cell with Ar (20 sccm) and introducing the anolyte (0.1 M K_2HPO_4 + 0.1 M KH_2PO_4 (pH 6.8)) (via the pump at 5 ml/min).
5. After a further 2 minutes introduce the catholyte and begin pre-reduction with Ar flow immediately (-226 µA cm^{-2}).
6. When the potential vs Ag/AgCl reaches ~-0.9 V *vs* Ag/AgCl (time depends on surface roughness), leave for a further half an hour and then begin CV in Ar.
7. CV in Ar between -0.5 V and -1.2 V vs NHE at 50 mV s^{-1} for at least 10 cycles until the CV has stabilized over a few crossings.
8. Hold at -0.5 V vs NHE and introduce CO, purging the cell for 30 minutes.
9. CV in CO between -0.5 V and -1.2 V *vs* NHE at 50 mV s-1 for 10 cycles.
10. Subtract the baseline and then integrate the area. The second method is preferred for the roughened surface and is described step by step below.
    a. Peak analyser
    b. Subtract baseline – manual
    c. Baseline mode - user defined, 2nd derivative
    d. Add points as shown below.

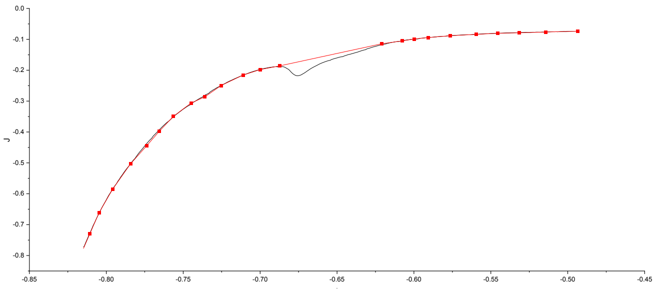

e. Plot the subtracted baseline curve

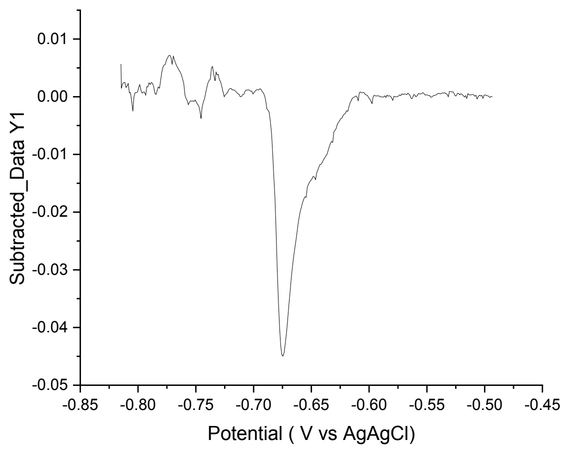

f. Integrate subtracted baseline curve using polygon area or peak analyser.

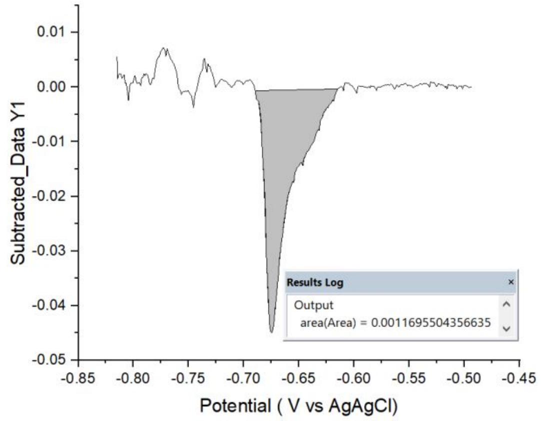

‘Potential hold’

1. Repeat steps 1-5 as in ‘CV integration’
2. Hold reduced pre-treated electrode at potential more than the displacement potential in the CV integration, whilst purging Ar at 20 sccm. This requires a CV beforehand to understand the correct potential to choose.
3. Wait until the current has stabilized. Here the potential hold in Ar was undertaken until the current had been stable, for more than 500s. Stable was defined as not climbing my more than 0.1 of a mA across the 7.56 cm^2 electrode as per Figure S10a.
4. CO was introduced to the cell and then left until the current had increased, then decreased past the initial stable current.
5. The area between the stable current, and the then decreasing line (as in Figure S10) was integrated to give the ‘potential hold’ measurement.

H-cell CO_2 reduction measurement

Electrochemical CO_2 reduction measurements were taken on the same electrodes in the same H cell (Figure S5 and S6) right after each CO displacement measurements. The electrolyte chamber was rinsed with 18.2 MΩ cm type 1 DI water for at least 5 times, before filled with 8 mL of 0.1 M $KHCO_3$ on each side. The electrolyte was prepared from 0.1 M KOH (potassium hydroxide hydrate, Merck Suprapur®, ≥99.995%) by purging with CO_2 until pH reaching 6.8. A pre-calibrated Ag/AgCl (Metrohm) and 0.5 mm thick Pt plate (Goodfellow Cambridge Limited, purity 99.95%) were used as the reference and counter electrode for all measurements. The cell was purged with 20 SCCM CO_2 for at least another 20 min for deaeration. Several CV cycles between -1 V and -1.5 V vs Ag/AgCl were taken until stabilized to fully reduce Cu. For each measurement, 1 h chronoamperometry at -1.000 V *vs* RHE were taken with 85% in situ ohmic drop compensation. The rest 15% was corrected manually after experiments. 20 SCCM CO_2 gas was purged into the cell during the chronoamperometry measurement.

Gas products were analysed by an on-line GC (SRI Multiple Gas Analyzer #5, equipped with a methanizer, TCD, and FID detector). H2 was measured by TCD, while all other gas products (including CO, CH_4, and ethylene) were detected by FID. After each chronoamperometry measurement starts, the cell was purged with 20 SCCM CO_2 for10 min until the first GC run. 3 GC runs were taken for each 1 h chronoamperometry CO_2 reduction test.

Liquid produces were analysed by 500 MHz ^{1}H-NMR (Bruker). Water suppression was conducted *via* a pre-saturation pulse sequence. 10 mM DMSO + 50 mM phenol in D_2O were added as the internal standards. Each NMR sample contains 12.3 µL of internal standards, 250 µL of electrolyte and 350 µL of D_2O. Both analyte and catholyte were analysed in case of the liquid product crossing over through the membrane.

EC-MS measurement

For the EC-MS measurement, polycrystalline Cu (5.0 mm OD x 4.0 mm thick, mirror polished, PINE) was used as the working electrode. The electrode underwent the same electropolishing process described in the main text prior to testing. A Pt mesh served as the counter electrode, and the same Ag/AgCl electrode (Metrohm) was used as the reference electrode. The electrolyte consisted of a pH 6.8 phosphate buffer prepared from 0.1 M K_2HPO_4 + 0.1 M KH_2PO_4, same as the CO displacement electrolyte. Further details regarding the cell geometry are provided below Figure S18. After assembling the cell and injecting the electrolyte, He was purged for deaeration with the flow rate of 1 mL min^{-1}. Meanwhile, cyclic voltammetry (CV) between -0.1 V and -0.6 V *vs* RHE with the scan rate of 50 mV s^{-1} was conducted until

stabilization, to remove the Cu surface oxidation layer, which usually takes around 15 cycles. Afterwards, gas was switched to CO with the same flow rate (1 mL min^{-1}), and another 15 cycles of CV were taken in CO. Finally, the CO reduction measurement was performed in the chronoamperometry mode. A series of potentials ranging from -0.96 V to -1.08 V *vs* NHE were applied, each for a duration of 1 minute. Between each potential step, the working electrode was held at -0.06 V *vs* NHE for 1 minute to allow the mass spectrometry (MS) signal to return to its baseline. H_2, CH_4, and C_2H_4 were measured *via* the mass spectrometer, through mass 2, 15, and 26, respectively. Only H_2 was detected and quantified, showing no CO reduction happening in 0.1 M K_2HPO_4 + 0.1 M KH_2PO_4 phosphate buffer under the potential window of interest in this work. The partial current density of H_2 can be calculated by:

$$I_{H_2} = \frac{n_{H_2} \cdot z_{H_2} \cdot F}{t}$$

where n_{H_2} is the number of moles of H_2 generated in the time span of t, z_{H_2} is the electron transferring number for H_2, I_{total} is the overall current that measured, and F is the faraday constant. n_{H_2} can be calculated by:

$$n_{H_2} = \frac{MS_{H_2} \cdot t}{SF_{H_2}}$$

where MS_{H_2} is the MS signal intensity of H_2, t is the time span, and SF_{H_2} is the sensitivity factor of H_2, which is the slope of the calibration curve. H_2 calibration was performed in the same setup, electrochemically, where glassy carbon disk (SIGRADUR G, 5.0 mm OD x 4.0 mm thick, 0.196 cm^2) was used as the working electrode. Different currents were applied to the working electrode, to allow the multi-points calibration. Figure S19 shows the calibration curve for H_2. The EC-MS results were analyzed *via* the open-source NumPy[1] and IXDAT[2] python package (available from https://github.com/ixdat).

Data in table format

Table S1. Roughness factors and normalised CO displacement charge of the 4 different Cu-based materials pretreated by; electropolishing, anodization and electrodeposition.

**Name of electrode**	**CO displacement peak ($mC\ cm^{-2}$)**	**±**	**Normalised CO Displacement charge**	**±**	**Potential hold peak (-0.85 V *vs* NHE) ($mC\ cm^{-2}$)**	**±**	**Potential hold normalised CO displacement charge**	**±**	**C_{dl} discharge ($mF\ cm^{-2}$)**	**C_{dl} roughness factor**	**±**
Electropolished	0.060	0.011	1.00	0.18	0.34	0.02	1.00	0.06	0.02	1.00	0.23
1.5 min anodized	0.118	0.010	1.96	0.16	-	-	-	-	0.16	6.86	1.20
Fully anodized	0.226	0.035	3.75	0.58	1.14	0.07	3.30	0.19	0.87	36.65	3.40
Electrodeposited	0.323	0.022	5.35	0.36	1.65	0.10	4.81	0.28	0.35	14.79	2.80

Table S2. FE, and geometric current density ($j_{geometric}$) for the pre-treated electrodes. A slight difference in potentials between each electrode was caused by ohmic drop correction.

		Electropolished (at -1.01 V *vs* RHE)	1.5 min anodized (at -1.03 V *vs* RHE)	Fully anodized (at -1.01 V *vs* RHE)	Electrodeposited (at -1.02 V *vs* RHE)
FE (%)	H_2	33.5	36.3	43.6	42.1
	CO	0.563	0.714	0.436	3.13
	CH_4	25.3	23.4	16.5	1.51
	C_2H_4	43.7	19.2	17.2	25.6
	$HCOO^-$	2.42	7.74	4.44	2.09
	EtOH	3.26	4.73	6.16	8.00
	CH_3COO^-	-	0.218	0.322	0.694
	n-PrOH	-	1.46	1.64	8.11
$j_{geometric}$ ($-mA\ cm^{-2}$)	H_2	0.683	3.119	6.344	7.512
	CO	0.016	0.061	0.063	0.558
	CH_4	0.882	2.012	2.407	0.269
	C_2H_4	1.417	1.646	2.511	4.581
	$HCOO^-$	0.070	0.665	0.646	0.373
	EtOH	0.095	0.407	0.897	1.429
	CH_3COO^-	-	0.019	0.045	0.124
	n-PrOH	-	0.125	0.238	1.448

Additional plots and figures

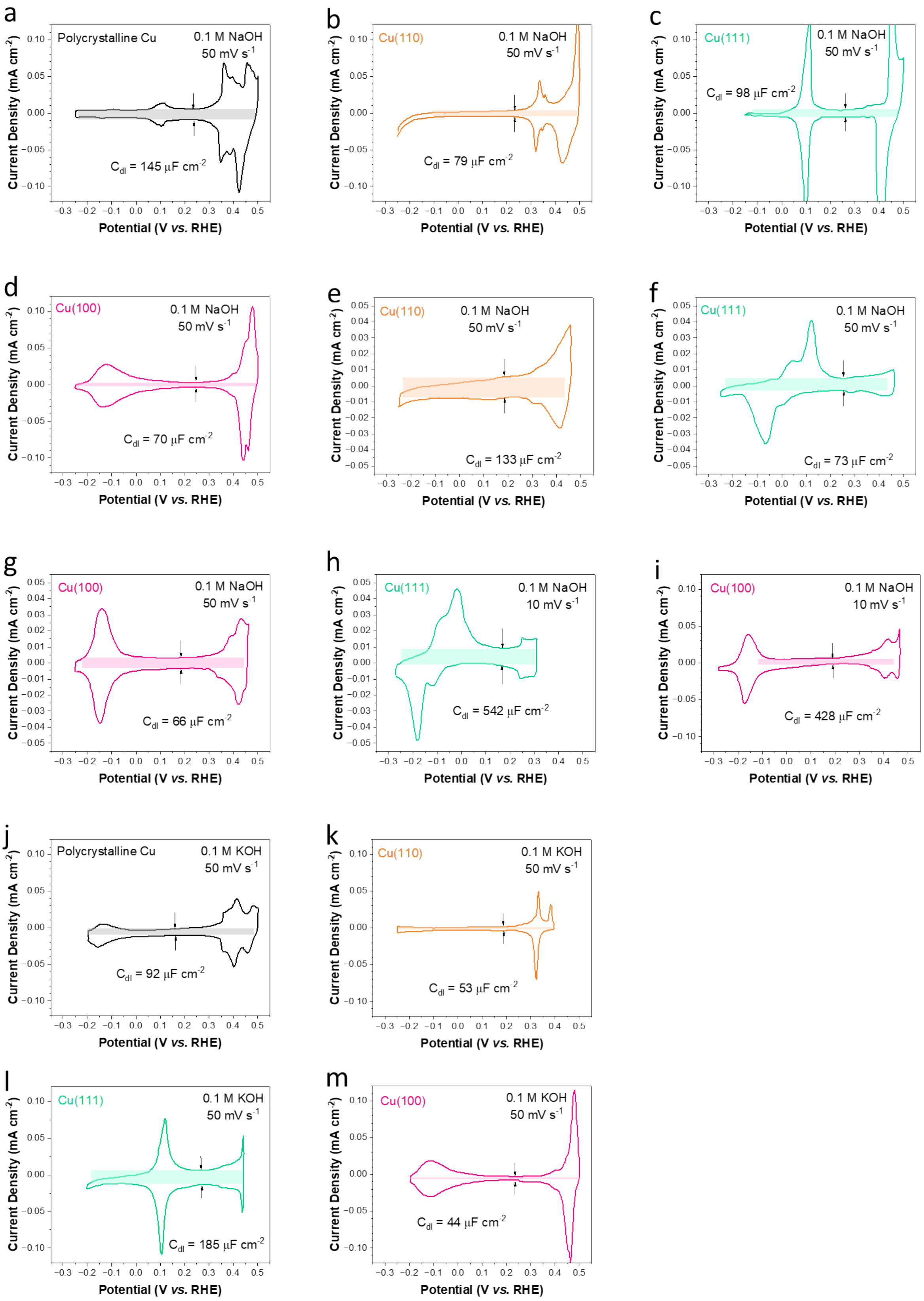

Figure S1. CV scans of Cu single crystals under alkaline conditions. Double layer capacitance (C_{dl}) was calculated by $C_{dl} = 0.5 * (j_{ox} – j_{red})$ / scan rate, where the coefficient 0.5 was used to separate the charging and discharging current. Experimental conditions were labelled on the figures. Data for (a)-(d) was taken and re-plotted from Raaijman *et al*[3]. Data for (e) – (g) was taken and re-plotted from Schouten *et al*[4]. Data for (h) and (i) was taken and re-plotted from Le Duff *et al*[5]. Date for (j) was taken and re-plotted from Engstfeld *et al*[6]. Data for (k) – (m) was taken and re-plotted from Tiwari *et al*[7].

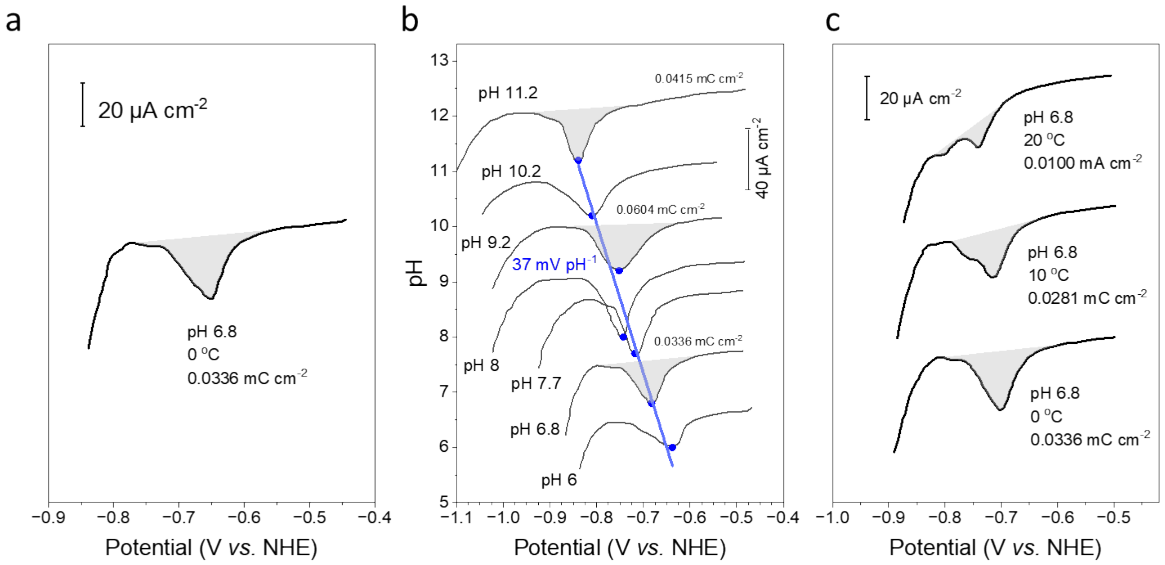

Figure S2. pH and temperature effect to the CO displacement. (a), Cathodic voltammogram of CO displacement on polycrystalline Cu with the scan rate of 50 mV s^{-1} in pH 6.8, 0.2 M phosphate buffer at 0 °C. (b), Cathodic voltammograms of CO displacement on polycrystalline Cu with the scan rate of 50 mV s^{-1} in 0.2 M phosphate buffers with different pHs at 0 °C. Solid blue line represents the relationship between the pH and CO displacement potential (37 mV pH^{-1}). (c), Cathodic voltammograms of CO displacement on polycrystalline Cu with the scan rate of 50 mV s^{-1} in pH 6.8, 0.2 M phosphate buffer at different temperatures. Data was taken and re-plotted from Hori *et al* [8].

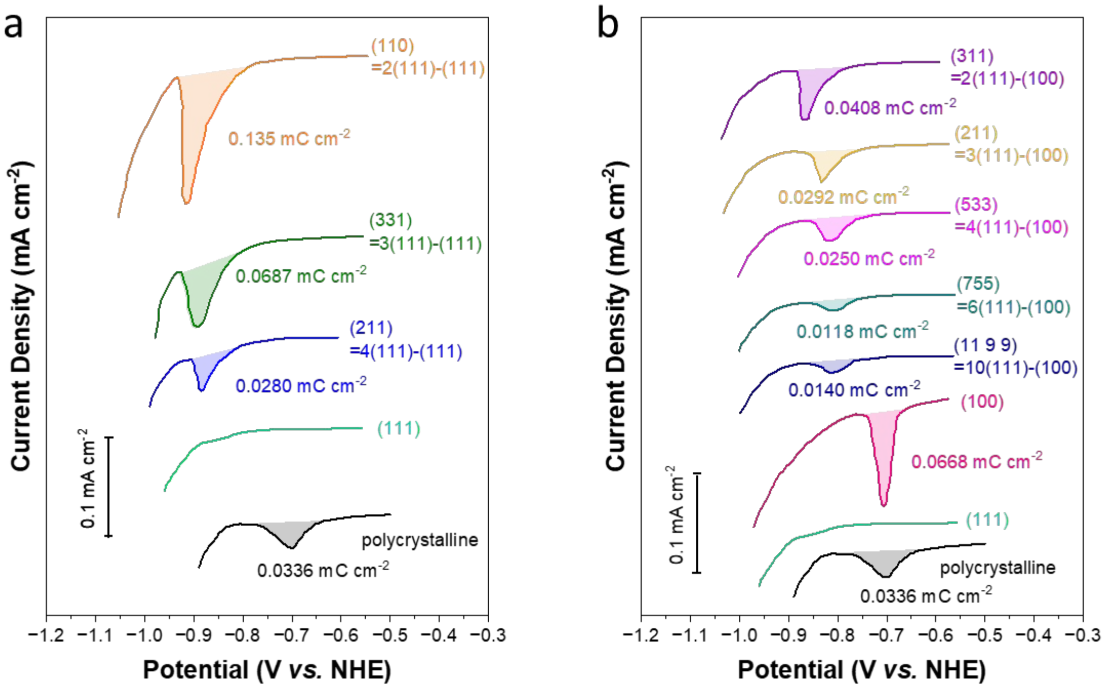

Figure S3. Facets dependency of CO displacement. CVs were taken on polycrystalline Cu and different Cu single crystals, with the scan rate of 50 mV s^{-1} in pH 6.8, CO saturated, 0.2 M phosphate buffer at 0 ^{o}C. Data were taken and re-plotted from Hori *et al* [9, 10].

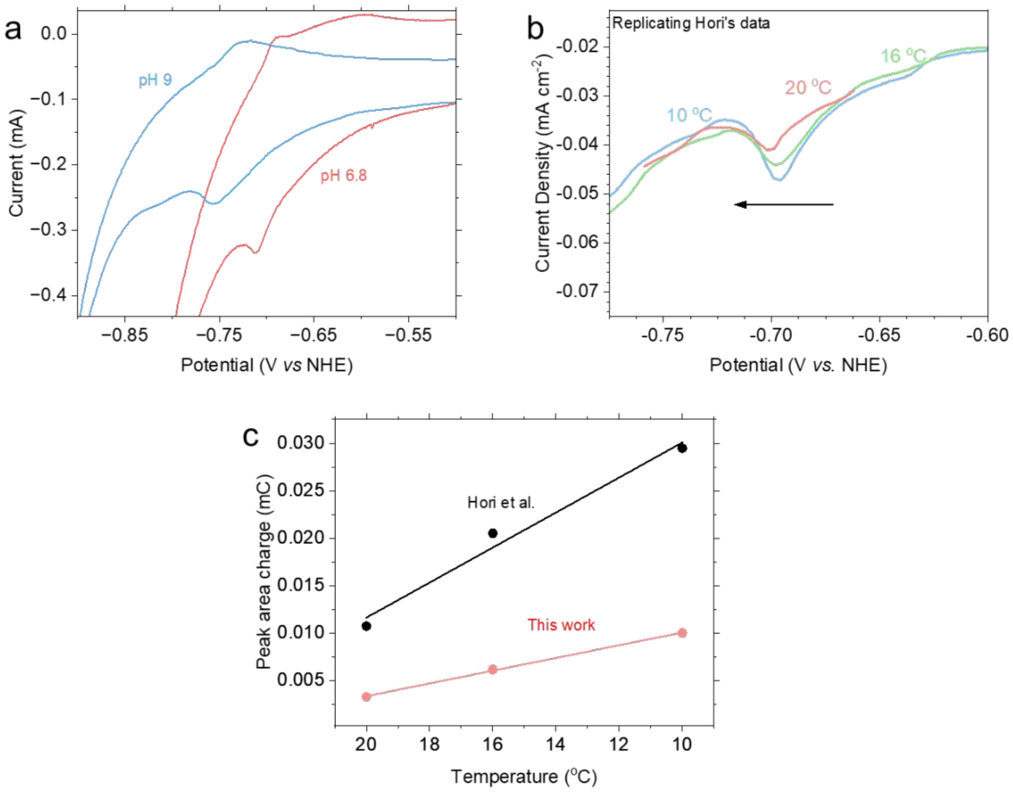

Figure S4. ***Replicating Hori et al. in our lab.*** (a), A CV scan of the CO displacement reaction at 50 mV s^{-1} in 0.1 M K_2HPO_4 + 0.1 M KH_2PO_4 pH 6.8 buffer (red) and 0.2 M K_2PO_4 buffer (blue). (b), An LSV scanned cathodically at 50 mV s^{-1} in 6.8 pH buffer (0.1 M K_2HPO_4 + 0.1 M KH_2PO_4) at different temperatures. (c), A graph showing the relationship between the temperature of the CO displacement reaction (CO exchanging with surface bound phosphate) and the peak area of charge for an electropolished copper foil with (200) as the preferred orientation in pH 6.8 buffer. Our work in red is compared to the work from Hori *et al* illustrated in black [8].

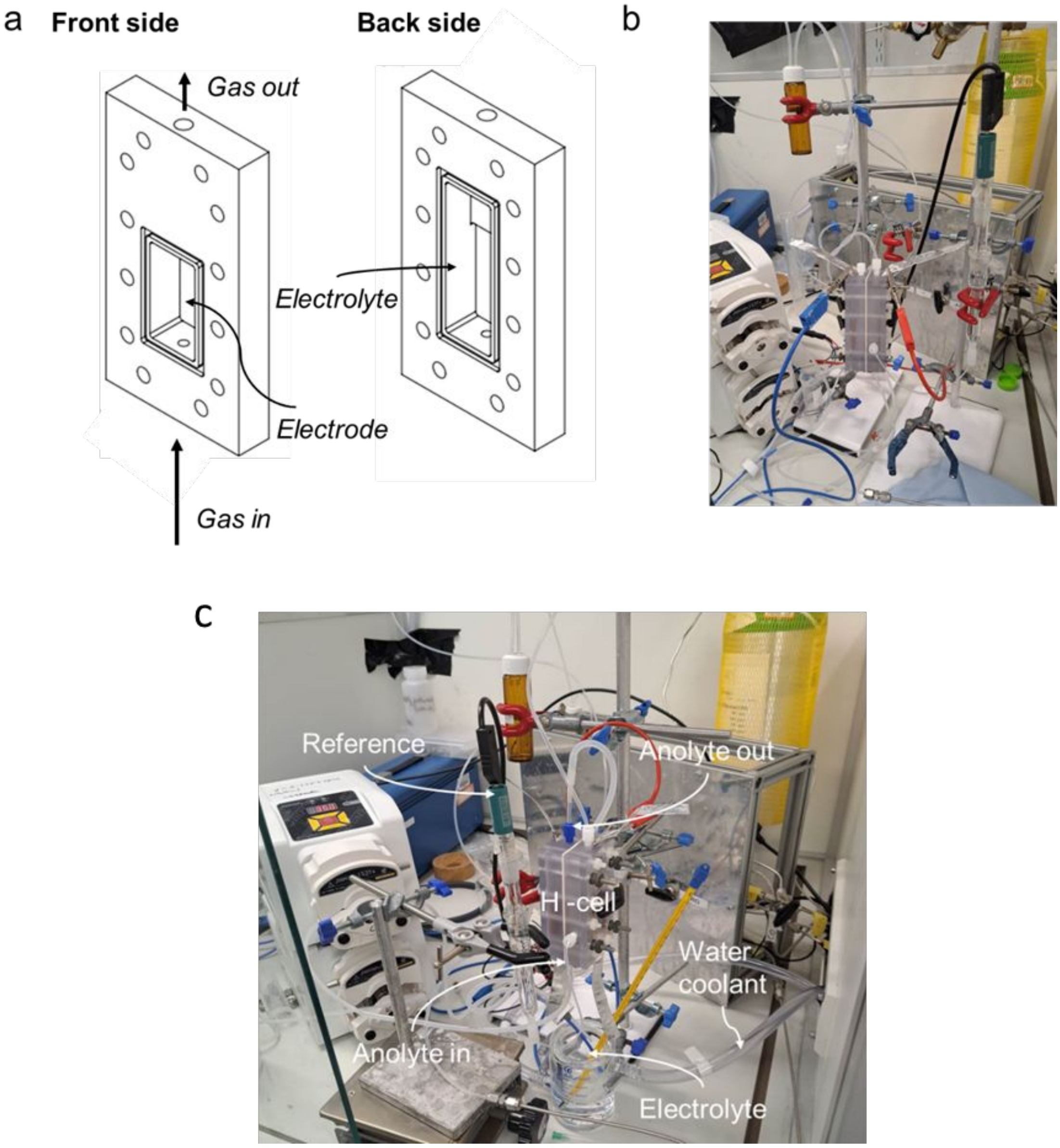

Figure S5. (a), Schematic illustration of the H-cell used in this work. (b), The H-cell assembled for testing a copper-based catalyst for CO_2RR, and double layer capacitance. (c) H-cell assembled for testing towards CO displacement. Cooled water flowed around the jacket beaker. The pump circulates the cooled anode into the H cell to cool the cathode side. The temperature (using a probe) was tracked in the catholyte, and a thermometer was placed in the anolyte to maintain the temperature of the reaction.

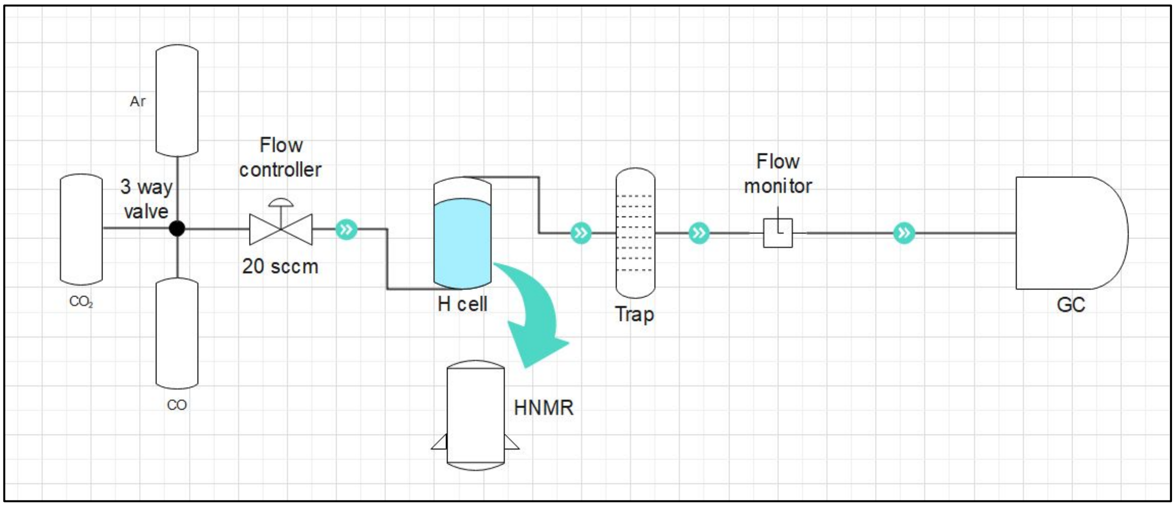

Figure S6. A schematic of the H-cell system for testing CO_2RR, double layer capacitance and CO displacement measurement.

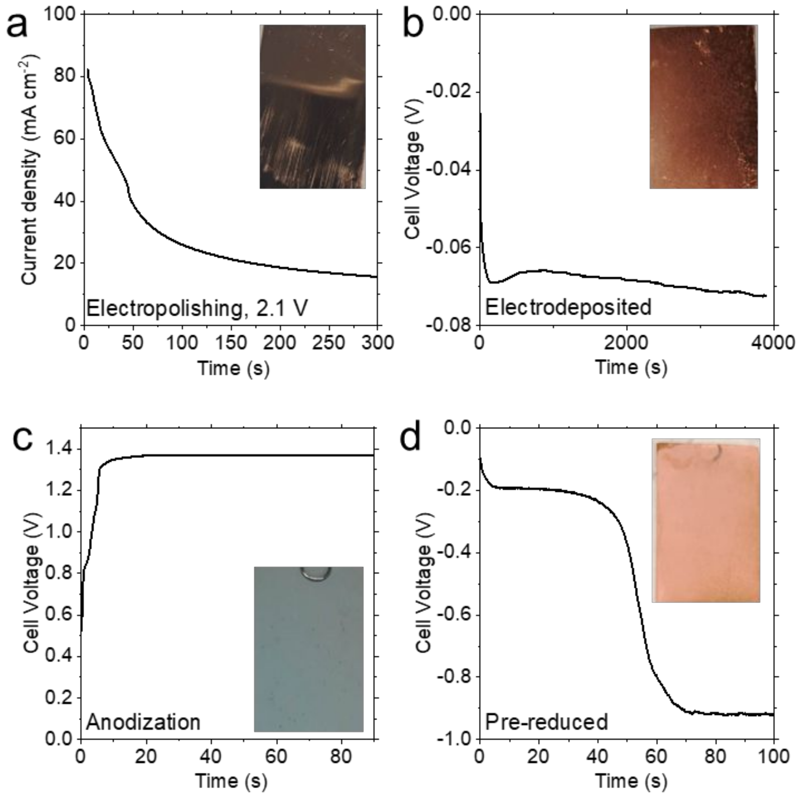

Figure S7. (a), Transient current curve for electropolishing the Cu foil in 5 M H_3PO_4, and its optical image; (b), Chronopotentiometry curve for the electrodeposition of Cu onto the electropolished Cu foil at -0.2 mA cm^{-2}, and its optical image; (c), Chronopotentiometry curve for the anodization of the electropolished copper foil. A current of 8 mA cm^{-2} was held for the anodization reaction in 3.0 M KOH[11, 12], and its optical image: (d), Chronopotentiometry curve for the in-situ reduction of anodised $Cu(OH)_2$ nanowires, and it's optical image. A current of -2 mA cm^{-2} was held for the reduction of the electrode in 0.1 M $KHCO_3$.

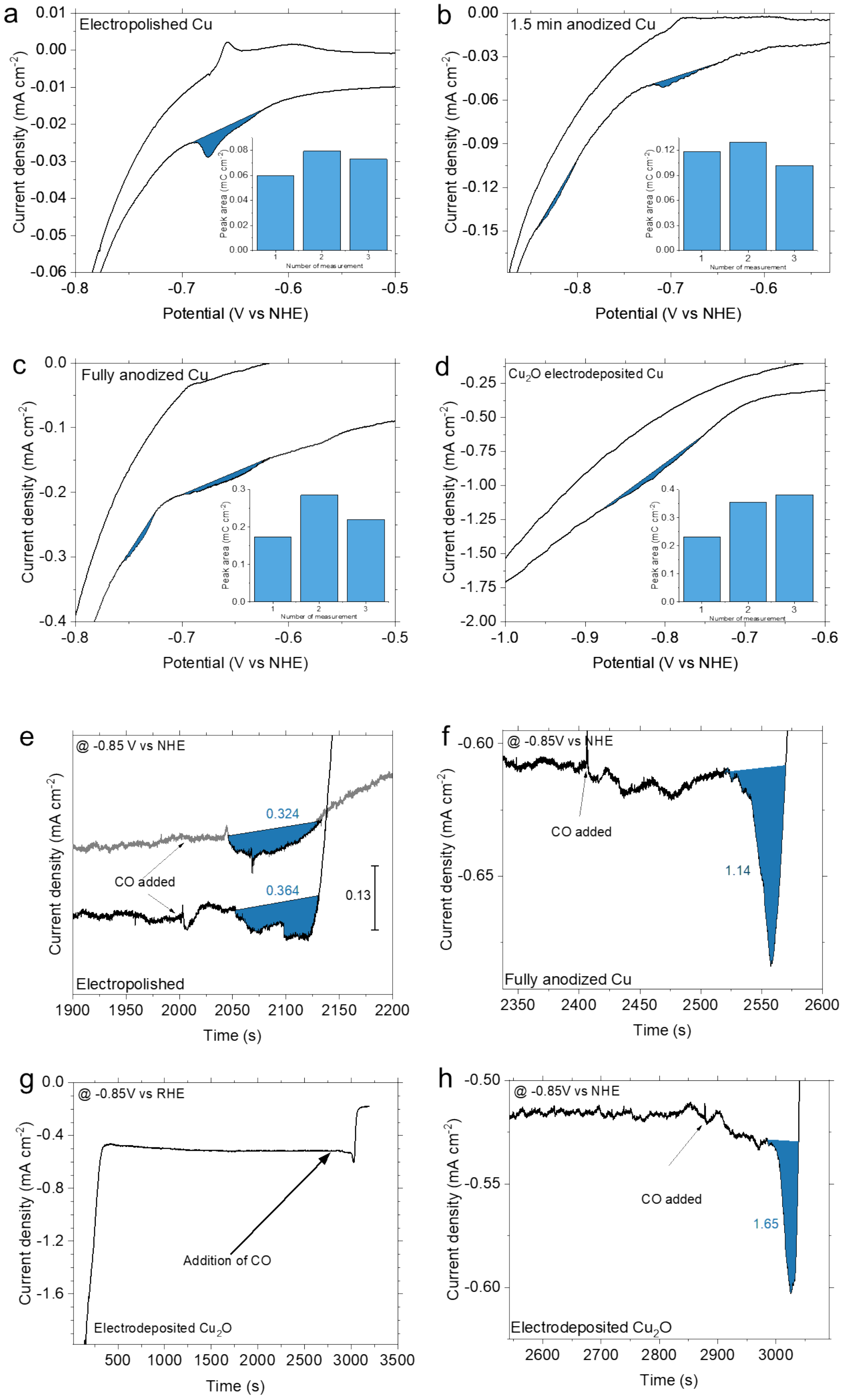

a
Electropolished Cu
Current density (mA cm-2)
Potential (V vs NHE)
Peak area (mC cm-2)
Number of measurement
b
1.5 min anodized Cu
c
Fully anodized Cu
d
Cu2O electrodeposited Cu
e
@ -0.85 V vs NHE
0.324
0.364
CO added
0.13
Electropolished
Time (s)
f
@ -0.85V vs NHE
CO added
1.14
Fully anodized Cu
g
@ -0.85V vs RHE
Addition of CO
Electrodeposited Cu2O
h
@ -0.85V vs NHE
CO added
1.65
Electrodeposited Cu2O

Figure S8. CO displacement CVs and the peak area values for the repeated measurements of (a) electropolished Cu, (b) 1.5-minute anodized copper foil, (c) fully anodized copper foil, and (d) Cu electrodeposited onto the copper foil between -0.5 V and -1.2 V *vs* NHE with a 50 mV s^{-1} scan rate at 10 ^{o}C in pH 6.8 phosphate buffer (0.1 M K_2HPO_4 + 0.1 M KH_2PO_4). Chronoamperometry curves of the potential hold measurements at -0.85 V *vs* NHE in pH 6.8 phosphate buffer (0.1 M K_2HPO_4 + 0.1 M KH_2PO_4) on (e) electropolished Cu, (f) fully anodized copper, (g) and (h) electrodeposited copper.

The multi-reduction CO displacement peaks at the cathodic CV scans in Figure S8 a to c are presumably caused by the different facets. Figure S8 e to h are transient current curves for the potential hold measurements. The electrodes were held at the potential slightly more negative than CO displacement (-0.85 V *vs* NHE), initially in Ar and then with the introduction of CO. The increase in current relates to the displacement of surface adsorbed phosphate ions. The potential, -0.85 V *vs* NHE, was selected based on both Hori's single crystal studies (Figure S3)[9, 10] and our own CV experiments (Figure S8 a-d). We avoided choosing a very negative potential to minimize the HER current effect. -0.85 V vs NHE should be more negative than, or at least similar with the onset potential for all the sites that can do CO displacement reaction.

Despite of the similar CO displacement charges, different magnitude of HER current suppression was observed in the repeating potential hold CO displacement experiment on the electropolished samples, as shown by Figure S8 e. The HER current was plotted against the normalised CO displacement charges, and poor linear relationship was found (Figure S20). This suggests using CO displacement results to interpretate HER activity of Cu would be inappropriate, as CO displacement probes only the CO_2 or CO reduction sites on Cu. The work from Engstfeld *et al*[6] showed that the same electropolishing method on different Cu foils could lead to very different CV behaviours. One possible explanation here is that electropolishing cleaned the surface, and created similar numbers of under-coordinated sites on both Cu electrodes. However, the rest terrace facets on both Cu electrodes after the same electropolishing method could be very different. As explained in the paper, only these under-coordinated sites might be active for CO_2 or CO reduction. CO displacement provides a method to quantify the amount of these under-coordinated sites. That might be the reason why similar CO displacement charges were obtained here. Due to the difference in the remaining terrace facets on both Cu electrodes, their HER behaviours might be different.

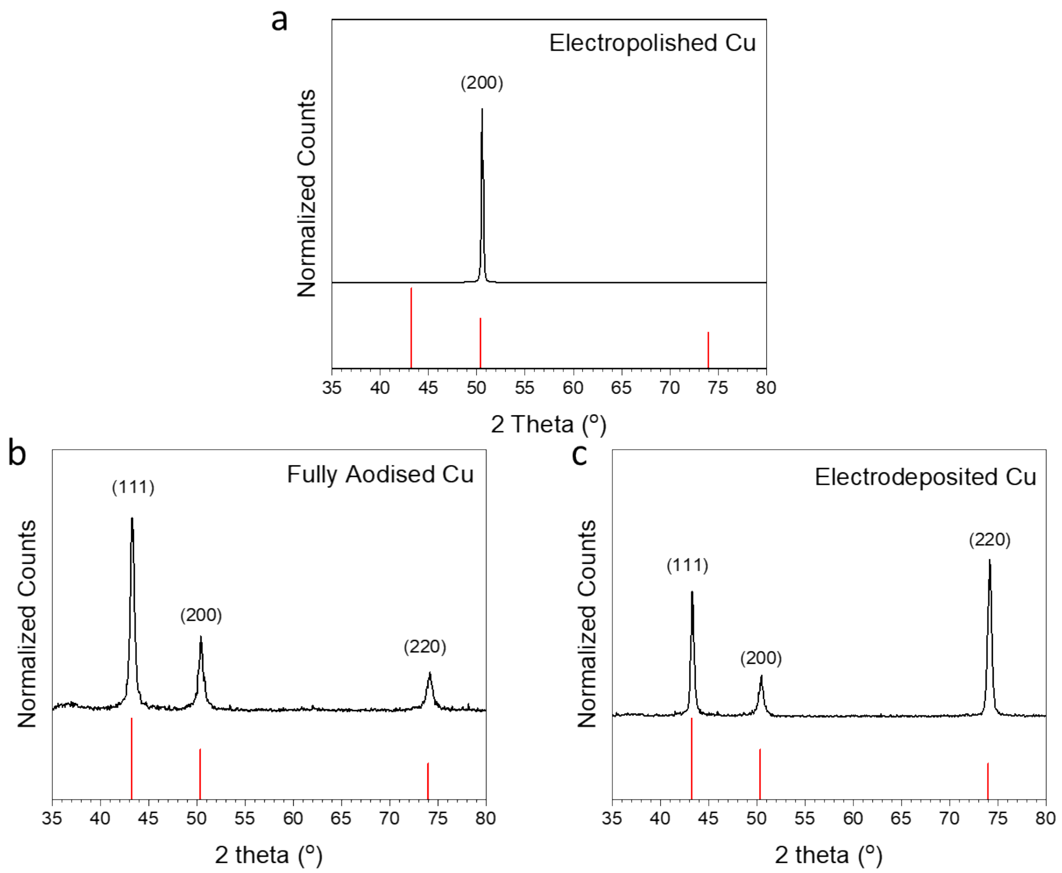

Figure S9. (a), XRD patterns of electropolished Cu plate. (b) and (c), Grazing Incidence XRD patterns of fully anodised Cu, and electrodeposited Cu, respectively, at the X-ray incidence angle of 0.5°, showing 100 nm of the surface. Both materials were electrochemically reduced in 0.1 M $KHCO_3$ at -226 μA cm^{-2} before diffraction. We wish to acknowledge the use of the EPSRC funded Physical Sciences Data-science Service hosted by the University of Southampton and STFC under grant number EP/S020357/1.

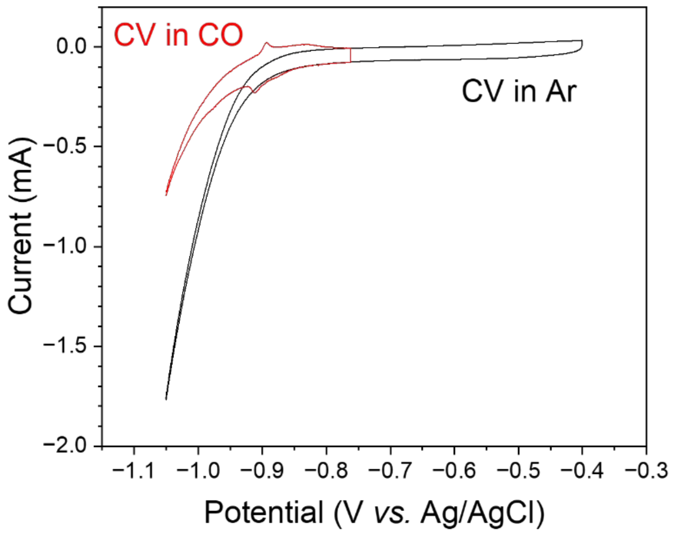

Figure S10. Comparison of CVs in just Ar, and CO for the electropolished Cu electrodes.

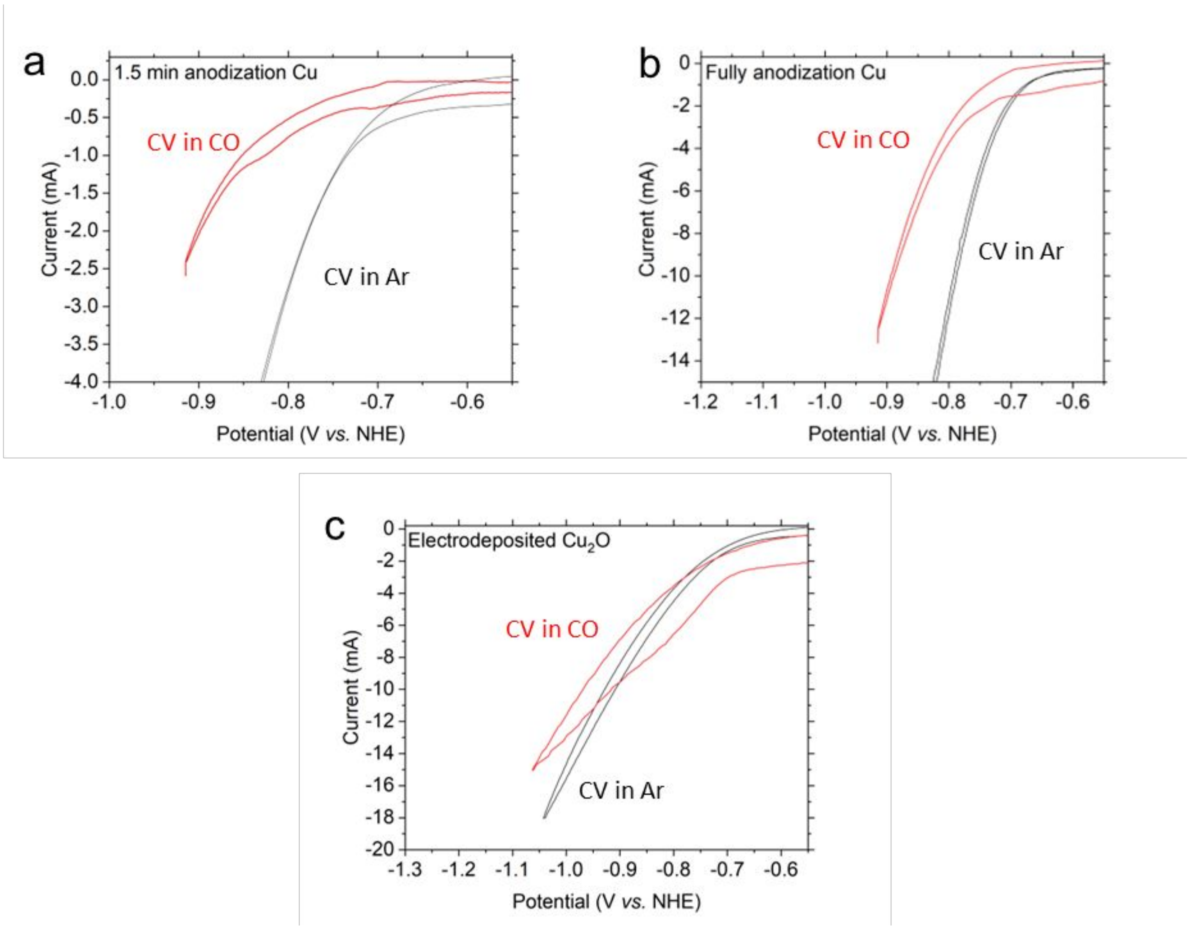

Figure S11. CVs illustrating that no other peak occurs at more negative than -0.9 V *vs* NHE for anodised and electropolished samples. The less HER current suppression by CO on the electrodeposited sample can also be explained by the different HER active sites distribution on the anodised samples and electrodeposited sample, similar as Figure S8 e. The H-cell possessed a geometric surface area of 7.56 cm^2 for the working electrode.

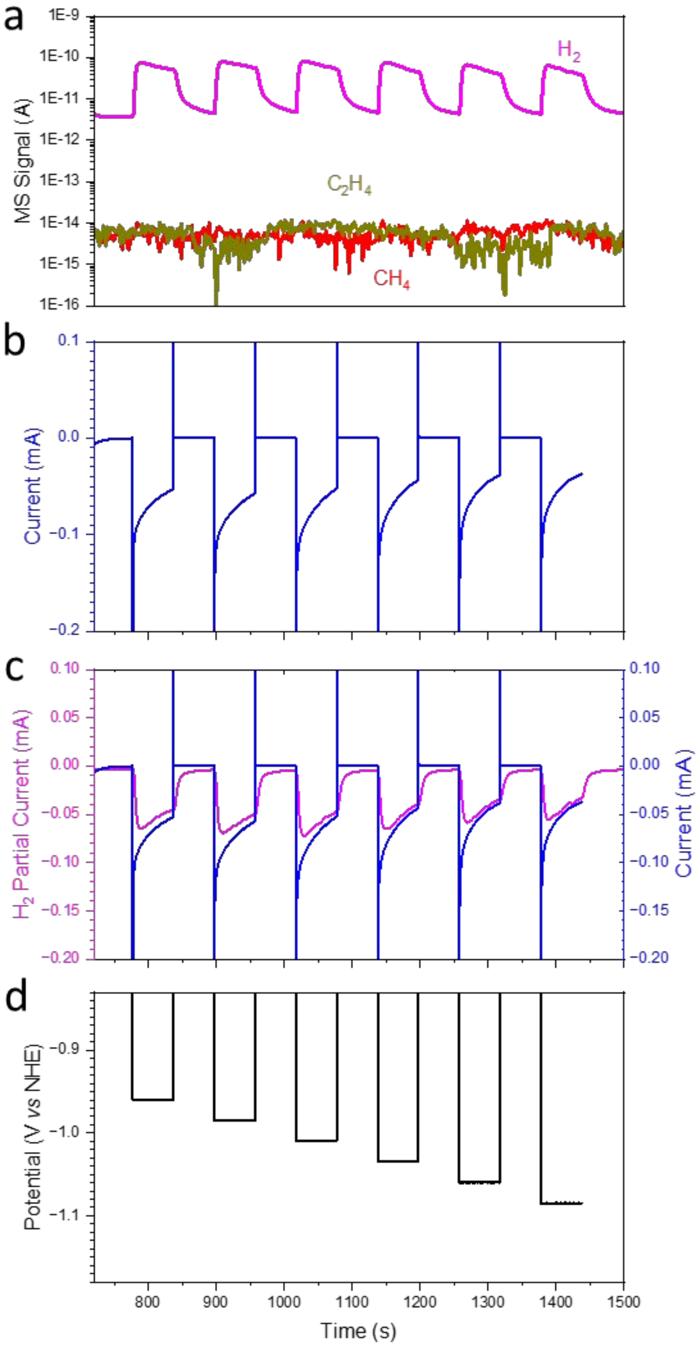

Figure S12. Electrochemistry mass spectra of H_2 (mass 2), CH_4 (mass 15), and C_2H_4 (mass 26) under different potentials in pH 6.8, 0.2 M phosphate buffer, showing that no CO reduction under the potential window of interest in this work. The EC-MS cell possessed a geometric surface area of 0.196 cm^2 for the working electrode.

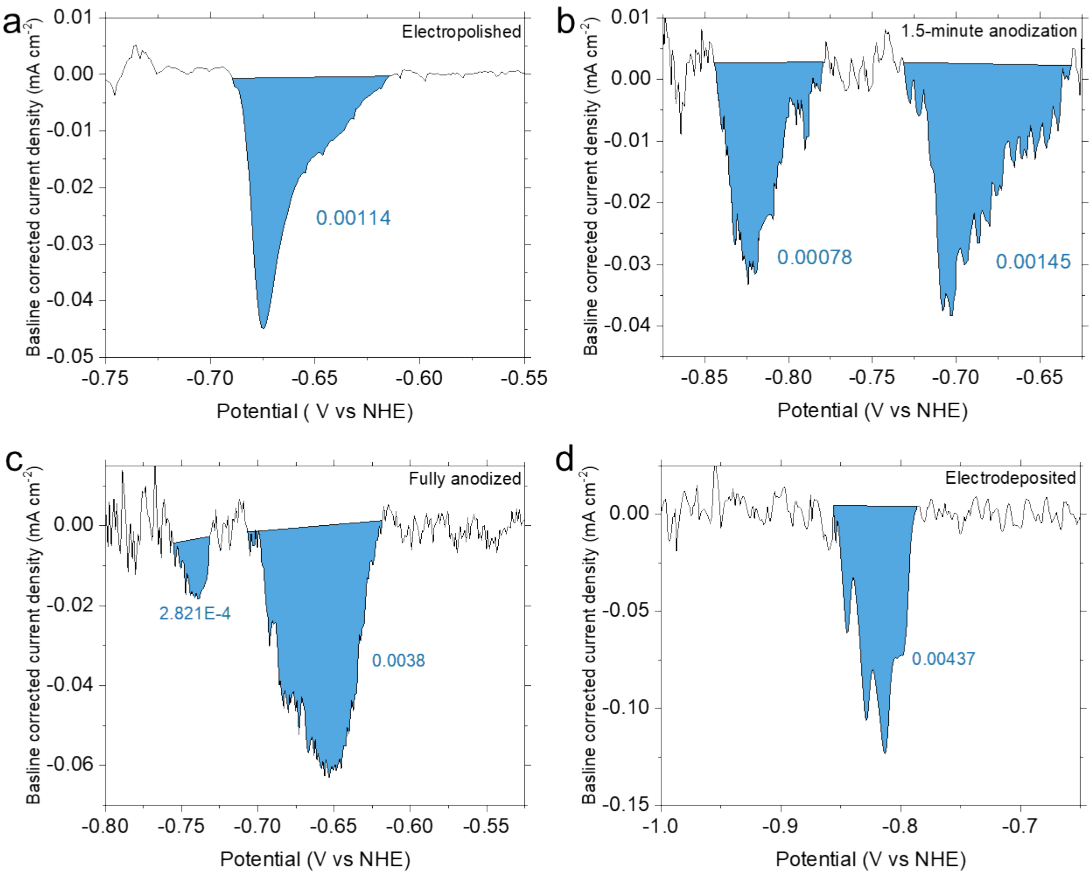

Figure S13. Illustrates the baseline corrected peak areas through user defined peak analyser via the Origin software. The process was repeated for the pre-treated electrodes; (a), electropolished copper foil, (b), 1.5-minute anodized copper foil, (c), fully anodized copper foil and (d), Cu electrodeposited onto the copper foil.

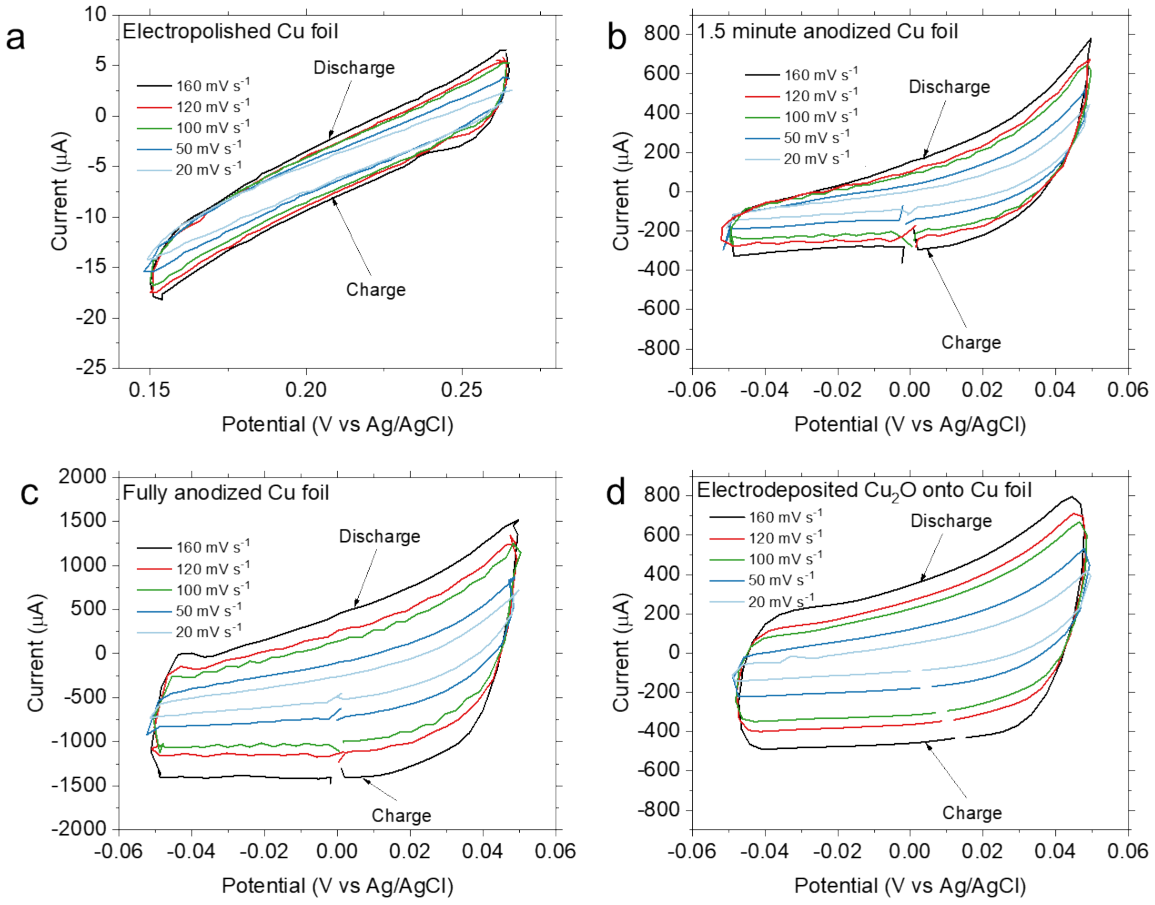

Figure S14. Double layer capacitance of all samples. A CV of the non-faradaic region in 0.1 M $HClO_4$ was taken at different scan rates.

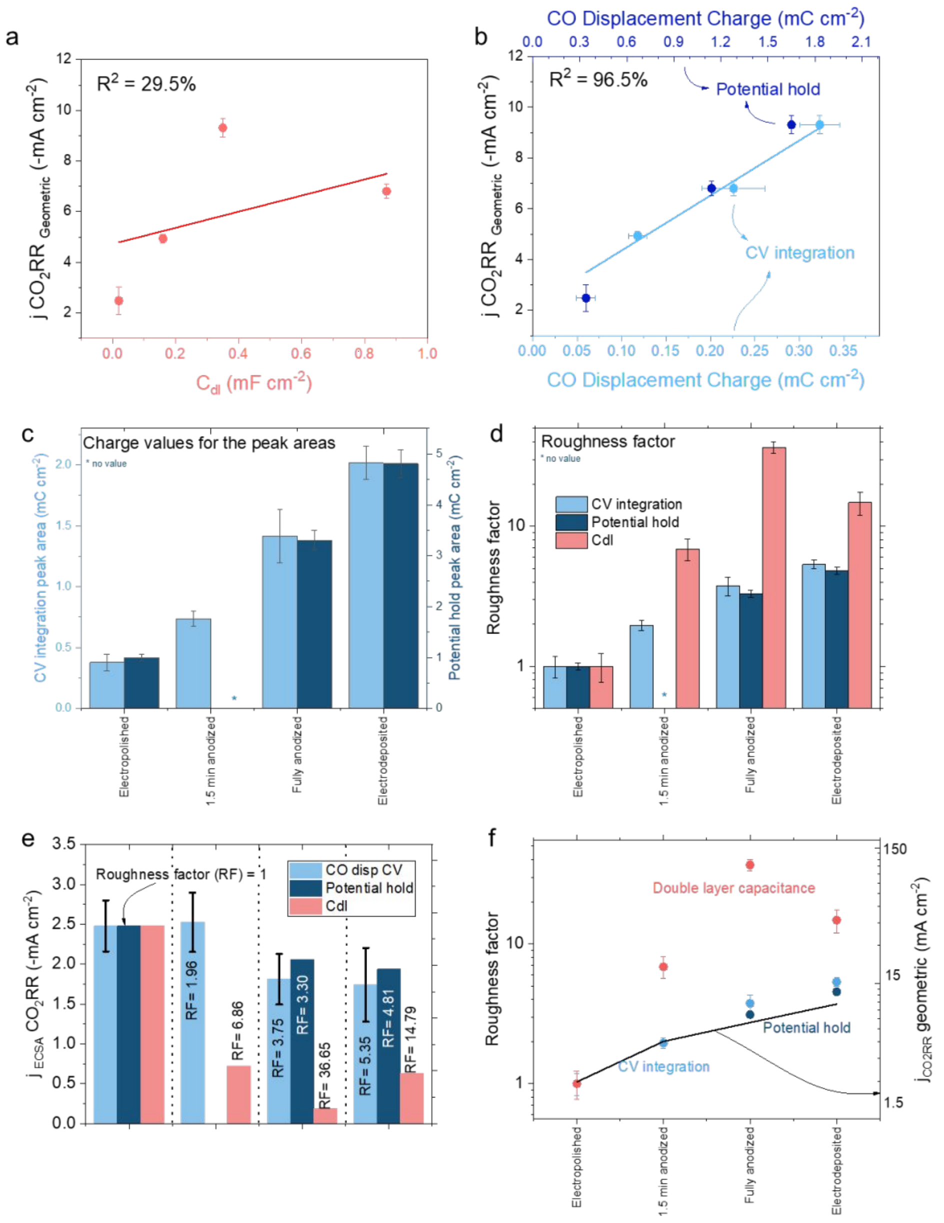

Figure S15. (a) Plot of CO_2RR partial current density normalized by geometric surface area against double layer capacitance; (b) Plot of CO_2RR partial current density normalized by geometric surface area against CO displacement charge measured by CV (light blue) and potential hold at -0.85 V *vs* NHE (dark blue); (c), Comparison between the CO displacement charges measured by CV and that measured by potential hold experiments; (d), Comparison of the roughness factors measured by CV CO displacement, potential hold CO displacement and double layer capacitance; (e), CO_2 reduction partial current densities normalised to active surface area calculated from the CV CO displacement (light blue), potential hold CO displacement (dark blue), and double layer capacitance (red) for the different pre-treated electrodes with the roughness factor (RF) labelled; (f), Co-plot of the roughness factors and CO_2 reduction partial current densities normalised to geometric surface area of 4 different materials.

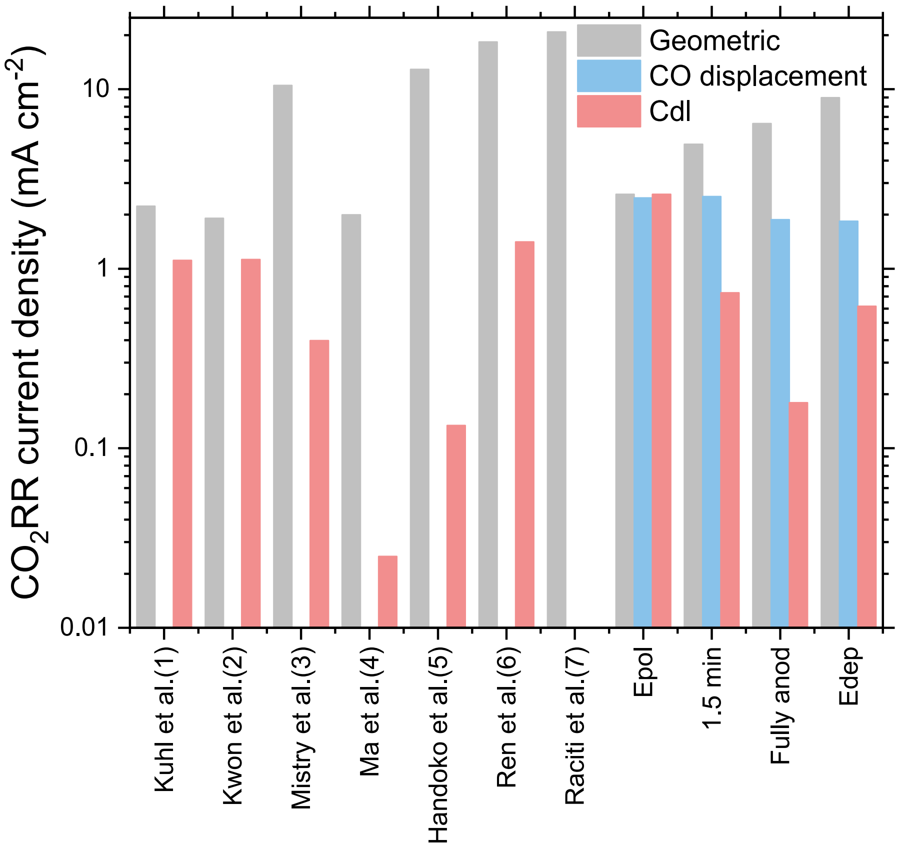

Figure S16. Comparison of geometric current density *vs* electrochemical active surface area current density across literature reproduced from Nitopi et al.[14]. Blue highlights the CO displacement current density reported in this work. Red the double layer capacitance normalized current density. (1)[15], (2)[16], (3)[17], (4)[18], (5)[19], (6)[20], (7)[21].

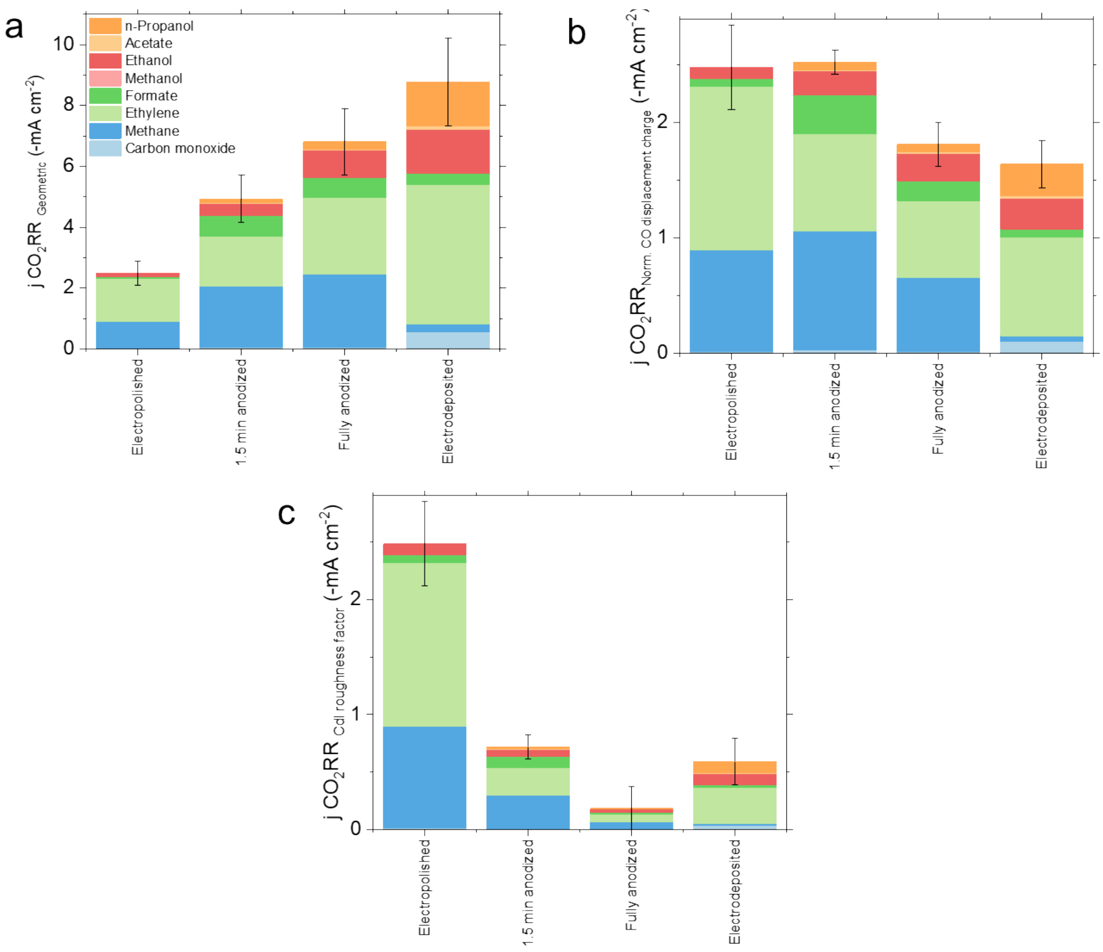

Figure S17. Partial current densities normalised to, (a) geometric surface area, (b), CO displacement charge and (c), double layer capacitance charge for the CO_2RR products produced from the different nanostructured electrodes. The products for the different normalized partial current density plots are shown in the legend of a.

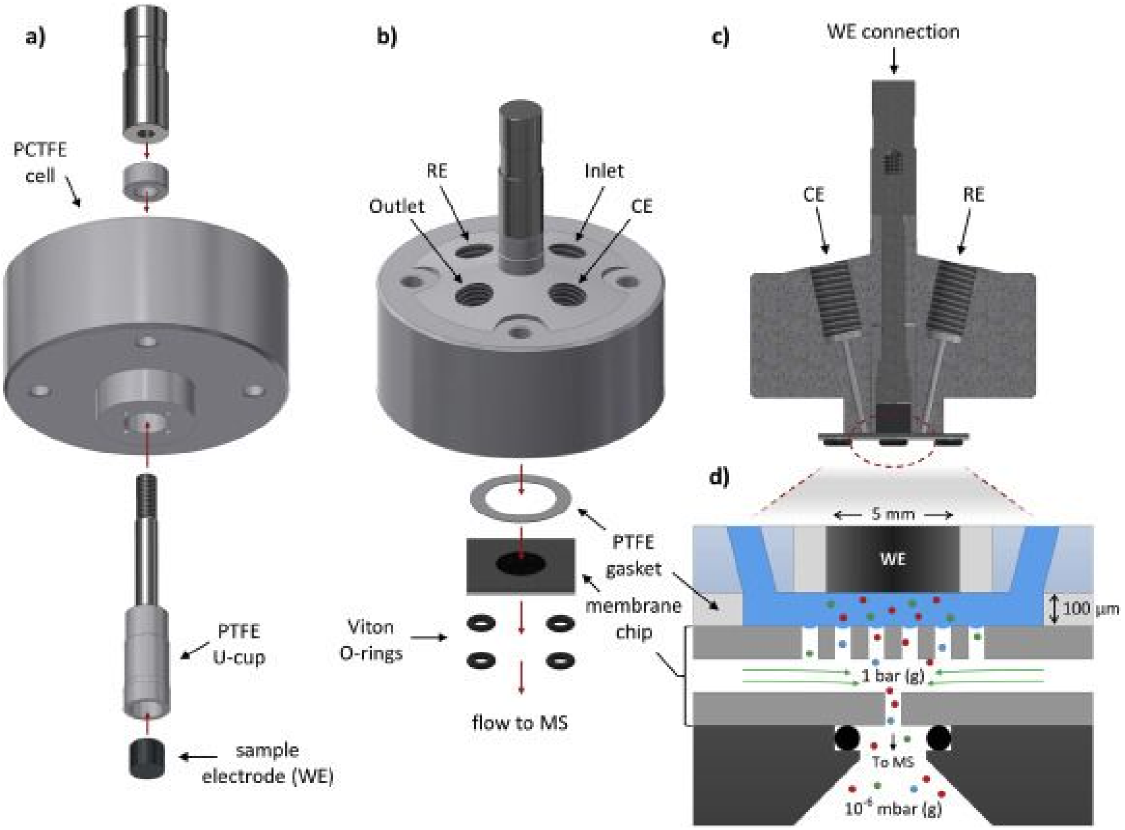

Figure S18. Schematic illustration of EC-MS. Reprinted from Trimarco *et al*[13].

Figure S18 shows the schematics of the electrochemistry mass spectrometer (EC-MS) used in this project. An electropolished polycrystalline Cu insert purchased from PINE was applied here as the working electrode. The PTFE U-cup purchased form PINE was used to mount the working electrode to the cell and as the connection to the potentiostat. 4 capillary tubes are connected to the electrolyte chamber, used for electrolyte inlet, outlet, placing the reference electrode, and counter electrode respectively. The cell is held 100 μm above the silicon-based porous membrane chip. The surface of the membrane chip is coated with a thin layer of hydrophobic polymer, which would prevent the aqueous electrolyte from pentration. The micro-channels in the membrane chip allows the purging of gaseous reactants like CO_2 and CO. Once the volatile molecules being produced on the working electrode surface, it will diffuse through the thin electrolyte layer (100 μm), evaporate on the membrane chip surface, and enter the vacuum chamber underneath the chip, followed by ionization and finally detected by the mass spectrometer. More detailed quantitative description of this process can be found from Trimarco et al[13].

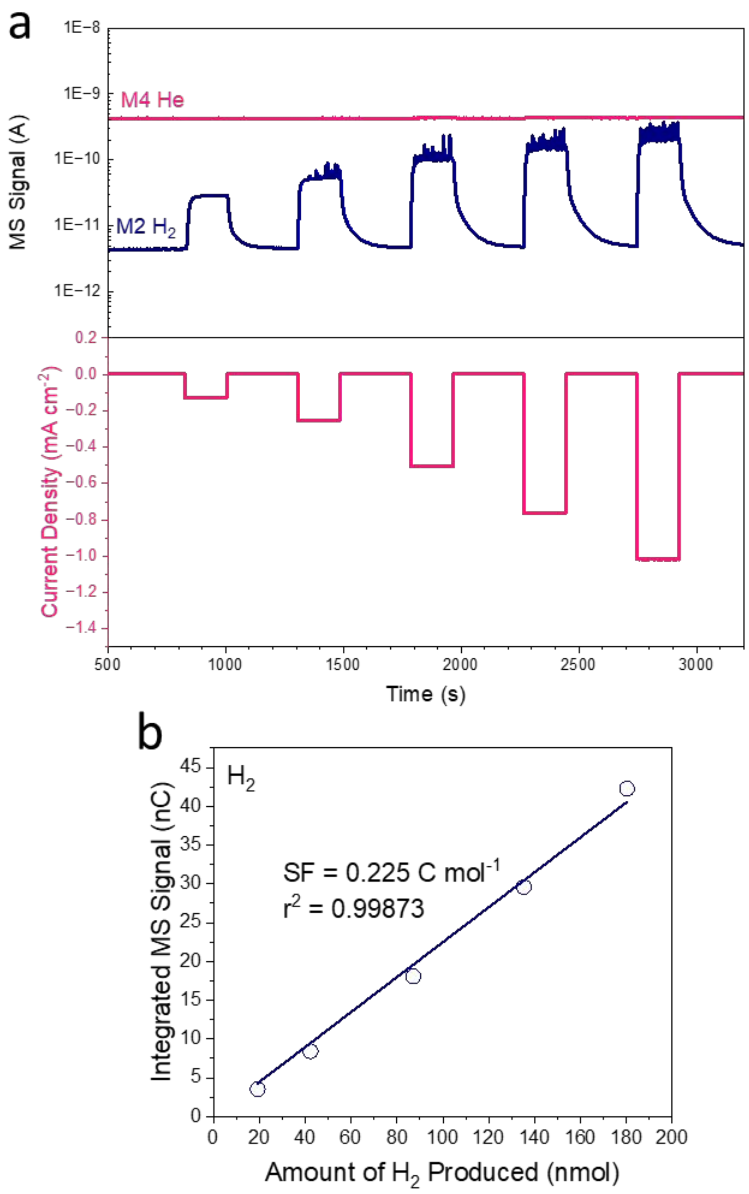

Figure S19. (a), EC-MS plot for H_2 calibration, and (b) Calibration curve for H_2.

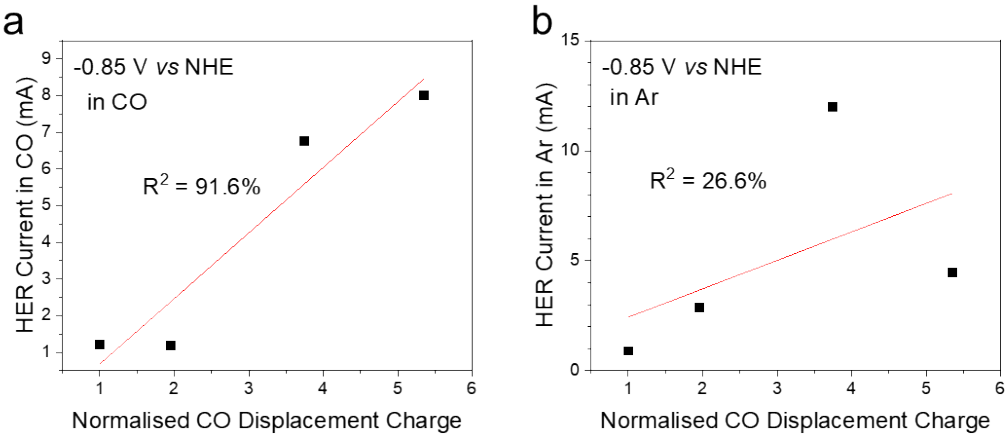

Figure S20. The plot of HER current in (a) Ar, and (b) in CO, against the normalised CO displacement charge. HER current value was taken from the cathodic scan of the CVs in Figure S8 a-d. The H-cell possessed a geometric surface area of 7.56 cm^2 for the working electrode. Poor linearity was obtained.

References

(1) Harris, C. R.; Millman, K. J.; Van Der Walt, S. J.; Gommers, R.; Virtanen, P.; Cournapeau, D.; Wieser, E.; Taylor, J.; Berg, S.; Smith, N. J. Array programming with NumPy. *nature* **2020**, *585* (7825), 357-362. DOI: 10.1038/s41586-020-2649-2.

(2) Scott, S. B.; Kibsgaard, J.; Vesborg, P. C.; Chorkendorff, I. Tracking oxygen atoms in electrochemical CO oxidation–Part I: Oxygen exchange via CO2 hydration. *Electrochimica Acta* **2021**, *374*, 137842. DOI: 10.1016/j.electacta.2021.137842.

(3) Raaijman, S. J.; Arulmozhi, N.; Koper, M. T. M. Morphological Stability of Copper Surfaces under Reducing Conditions. *ACS Applied Materials & Interfaces* **2021**, *13* (41), 48730-48744. DOI: 10.1021/acsami.1c13989.

(4) P. Schouten, K. J.; Gallent, E. P.; Koper, M. T. M. The electrochemical characterization of copper single-crystal electrodes in alkaline media. *Journal of Electroanalytical Chemistry* **2013**, *699*, 6-9. DOI: 10.1016/j.jelechem.2013.03.018.

(5) Le Duff, C. S.; Lawrence, M. J.; Rodriguez, P. Role of the adsorbed oxygen species in the selective electrochemical reduction of CO2 to alcohols and carbonyls on copper electrodes. *Angewandte Chemie* **2017**, *129* (42), 13099-13104. DOI: 10.1002/anie.201706463.

(6) Engstfeld, A. K.; Maagaard, T.; Horch, S.; Chorkendorff, I.; Stephens, I. E. L. Polycrystalline and Single-Crystal Cu Electrodes: Influence of Experimental Conditions on the Electrochemical Properties in Alkaline Media. *Chemistry – A European Journal* **2018**, *24* (67), 17743-17755. DOI: 10.1002/chem.201803418.

(7) Tiwari, A.; Heenen, H. H.; Bjørnlund, A. S.; Maagaard, T.; Cho, E.; Chorkendorff, I.; Kristoffersen, H. H.; Chan, K.; Horch, S. Fingerprint Voltammograms of Copper Single Crystals under Alkaline Conditions: A Fundamental Mechanistic Analysis. *The Journal of Physical Chemistry Letters* **2020**, *11* (4), 1450-1455. DOI: 10.1021/acs.jpclett.9b03728.

(8) Hori, Y.; Murata, A.; Tsukamoto, T.; Wakebe, H.; Koga, O.; Yamazaki, H. Adsoprtion of carbon monoxide at a copper electrode accompanied by electron transfer observed by voltammetry and IR spectroscopy. *Electrochimica Acta* **1994**, *39* (17), 2495-2500. DOI: 10.1016/0013-4686(94)00259-2.
(9) Hori, Y.; Wakebe, H.; Tsukamoto, T.; Koga, O. Adsorption of CO accompanied with simultaneous charge transfer on copper single crystal electrodes related with electrochemical reduction of CO2 to hydrocarbons. *Surface Science* **1995**, *335*, 258-263. DOI: 10.1016/0039-6028(95)00441-6.
(10) Koga, O.; Teruya, S.; Matsuda, K.; Minami, M.; Hoshi, N.; Hori, Y. Infrared spectroscopic and voltammetric study of adsorbed CO on stepped surfaces of copper monocrystalline electrodes. *Electrochimica Acta* **2005**, *50* (12), 2475-2485. DOI: 10.1016/j.electacta.2004.10.076.
(11) Luo, J.; Steier, L.; Son, M.-K.; Schreier, M.; Mayer, M. T.; Grätzel, M. Cu2O Nanowire Photocathodes for Efficient and Durable Solar Water Splitting. *Nano Letters* **2016**, *16* (3), 1848-1857. DOI: 10.1021/acs.nanolett.5b04929.
(12) Schreier, M.; Héroguel, F.; Steier, L.; Ahmad, S.; Luterbacher, J. S.; Mayer, M. T.; Luo, J.; Grätzel, M. Solar conversion of CO2 to CO using Earth-abundant electrocatalysts prepared by atomic layer modification of CuO. *Nature Energy* **2017**, *2* (7), 17087. DOI: 10.1038/nenergy.2017.87.
(13) Trimarco, D. B.; Scott, S. B.; Thilsted, A. H.; Pan, J. Y.; Pedersen, T.; Hansen, O.; Chorkendorff, I.; Vesborg, P. C. K. Enabling real-time detection of electrochemical desorption phenomena with sub-monolayer sensitivity. *Electrochimica Acta* **2018**, *268*, 520-530. DOI: 10.1016/j.electacta.2018.02.060.
(14) Nitopi, S.; Bertheussen, E.; Scott, S. B.; Liu, X.; Engstfeld, A. K.; Horch, S.; Seger, B.; Stephens, I. E. L.; Chan, K.; Hahn, C.; et al. Progress and Perspectives of Electrochemical CO2 Reduction on Copper in Aqueous Electrolyte. *Chemical Reviews* **2019**, *119* (12), 7610-7672. DOI: 10.1021/acs.chemrev.8b00705.
(15) Kuhl, K. P.; Cave, E. R.; Abram, D. N.; Jaramillo, T. F. New insights into the electrochemical reduction of carbon dioxide on metallic copper surfaces. *Energy & Environmental Science* **2012**, *5* (5), 7050-7059. DOI: 10.1039/C2EE21234J.
(16) Kwon, Y.; Lum, Y.; Clark, E. L.; Ager, J. W.; Bell, A. T. CO2 Electroreduction with Enhanced Ethylene and Ethanol Selectivity by Nanostructuring Polycrystalline Copper. *ChemElectroChem* **2016**, *3* (6), 1012-1019. DOI: 10.1002/celc.201600068.
(17) Mistry, H.; Varela, A. S.; Bonifacio, C. S.; Zegkinoglou, I.; Sinev, I.; Choi, Y.-W.; Kisslinger, K.; Stach, E. A.; Yang, J. C.; Strasser, P.; et al. Highly selective plasma-activated copper catalysts for carbon dioxide reduction to ethylene. *Nature Communications* **2016**, *7* (1), 12123. DOI: 10.1038/ncomms12123.
(18) Ma, M.; Djanashvili, K.; Smith, W. A. Selective electrochemical reduction of CO2 to CO on CuO-derived Cu nanowires. *Physical Chemistry Chemical Physics* **2015**, *17* (32), 20861-20867. DOI: 10.1039/C5CP03559G.
(19) Handoko, A. D.; Chan, K. W.; Yeo, B. S. –CH3 Mediated Pathway for the Electroreduction of CO2 to Ethane and Ethanol on Thick Oxide-Derived Copper Catalysts at Low Overpotentials. *ACS Energy Letters* **2017**, *2* (9), 2103-2109. DOI: 10.1021/acsenergylett.7b00514.
(20) Ren, D.; Wong, N. T.; Handoko, A. D.; Huang, Y.; Yeo, B. S. Mechanistic Insights into the Enhanced Activity and Stability of Agglomerated Cu Nanocrystals for the Electrochemical Reduction of Carbon Dioxide to n-Propanol. *The Journal of Physical Chemistry Letters* **2016**, *7* (1), 20-24. DOI: 10.1021/acs.jpclett.5b02554.
(21) Raciti, D.; Livi, K. J.; Wang, C. Highly Dense Cu Nanowires for Low-Overpotential CO2 Reduction. *Nano Letters* **2015**, *15* (10), 6829-6835. DOI: 10.1021/acs.nanolett.5b03298.
